# Spinal glycine receptor alpha 1 coordinates startle behavior through a cell-type specific mechanism

**DOI:** 10.7150/ijbs.132688

**Published:** 2026-07-13

**Authors:** Shoupeng Wei, Jiyi Xu, Shao-Rui Chen, Yuying Huang, Zev Jarrett, Grzegorz Godlewski, Janos Paloczi, Pal Pacher, Hui-Lin Pan, Yan Xu, David M. Lovinger, Li Zhang

**Affiliations:** 1 Laboratory for Integrative Neuroscience, National Institute on Alcohol Abuse and Alcoholism, National Institutes of Health, Bethesda, MD 20892, USA.; 2 Department of Anesthesiology and Perioperative Medicine, University of Pittsburgh, Pittsburgh, PA 15260, USA.; 3 Department of Anesthesiology and Perioperative Medicine, The University of Texas MD Anderson Cancer Center, Houston, TX 77030, USA.; 4 Laboratory of Physiologic Studies, National Institute on Alcohol Abuse and Alcoholism, National Institutes of Health, Bethesda, MD 20892, USA.; 5 Laboratory of Cardiovascular Physiology and Tissue Injury, National Institutes of Health/NIAAA, Bethesda, MD 20892, USA.

**Keywords:** startle, spinal cord, interneuron, glycine receptor alpha 1, GlyRα1, GlyT2, ChAT, CamK2α, vGluT2

## Abstract

The spinal cord critically regulates startle responses through local excitatory and inhibitory circuits. The glycine receptor alpha 1 subunit (GlyRα1) mediates the primary inhibitory neurotransmission in the spinal cord, however, the contribution of spinal glycinergic inhibition to startle regulation remains poorly understood. Here, we show that GlyRα1 controls startle behavior in a cell-type- and region-specific manner. GlyRα1 deletion in ChAT-positive spinal neurons markedly enhances startle responses and c-Fos activation in spinal motor neurons as well as excitatory interneurons. In contrast, GlyRα1 deletion in inhibitory interneurons suppresses startle behavior while selectively increasing spinal c-Fos activation in inhibitory but not excitatory interneurons. Consistent with these findings, intraspinal AAV-Cre induced deletion of GlyRα1 without cell-type specificity increases startle responses, whereas deletion from spinal CamK2α-positive cells, which predominantly overlap with glycinergic inhibitory interneurons, attenuates startle reflexes. By contrast, deletion of GlyRα1 from brainstem RtTg CamK2α-positive cells, which are predominantly glutamatergic, enhances startle responses. GlyRα1 deficiency in glycinergic neurons reduces the amplitude of puff-applied glycine-elicited currents without affecting synaptic inhibitory and excitatory neurotransmission in the spinal cord. Together, these findings identify spinal GlyRα1 subunits as a cell-type specific regulator of startle behavior and reveal opposing contributions of GlyRα1 signaling in spinal inhibitory interneurons and motor/excitatory output pathways.

## Introduction

The primary startle response (SR) is a natural reflex elicited by a sudden intensive acoustic or tactile stimulus 1-3. An abnormal startle phenotype in the clinic is considered as a hallmark of neuropsychiatric disorders 4. The formation of acoustic startle response (ASR) involves auditory nerve fibers, cochlear root neurons, the caudal pontine reticular nucleus (PnC) and brainstem reticulotegmental (RtTg) pathways, which relay signals to interneurons, control activity of spinal motor neurons and modulate startle response 5, 6. In contrast to our understanding of the brainstem circuits that mediate the startle response, the role of the spinal cord in coordinating the startle reflex remains largely unclear. The spinal cord receives the descending projections from both the brainstem and motor cortex, integrating and executing involuntary and voluntary motor commands at the level of motor neurons through a balance of excitatory and inhibitory neurotransmission. These processes within the spinal cord are tightly regulated by local interneuron populations, such as inhibitory (glycinergic and GABAergic) neurons, excitatory (glutamatergic) and cholinergic motoneurons 7. Until recently, relatively little has been known about how these neuronal populations coordinate the startle reflex signals from the descending brainstem pathways to generate precise motor outputs.

Glycine receptor α1 subunits (GlyRα1) mediate the majority of inhibitory neurotransmission in the spinal cord 8. Numerous naturally occurring point-mutations in GlyRα1 result in loss of function and exaggerated startle response in humans and animals 3, 9-13. Although rare, this disease is characterized by excessive startle reactions to unexpected auditory and tactile stimuli followed by muscle stiffness 3, 14. Both presynaptic and postsynaptic GlyRα1 deficiency leads to hyperekplexia, and a similar phenotype is observed with dysfunction of the neuronal glycine transporter 2 (GlyT2) 3, 15-19. There is also evidence suggesting that extrasynaptic glycine receptors (GlyRs) are involved in the control of startle circuit and plasticity 20. While brainstem GlyRα1 function is the primary focus of startle research, the role of spinal GlyRα1 in startle response remains unreported. Although GlyRα1 subunits are abundantly expressed in most brainstem and spinal neuron classes in adulthood, the cell-type specific mechanisms by which GlyRα1 subunits regulate the startle reflex remain poorly understood 8, 21. To explore the cellular mechanisms by which GlyRα1 subunits regulate the startle response through its inhibition and disinhibition of excitatory and inhibitory interneurons in the spinal cord, we generated transgenic mice with either global or spinal cord cell-type specific deficiency in GlyRα1 and examined their startle response to acoustic and tactile (air-puff) stimuli. Mice with cell-type and spinal-cord specific deletion of GlyRα1 exhibited the opposing effects on startle response. Our findings further indicate that spinal GlyRα1 subunits regulate acoustic or tactile stimuli-induced startle responses through the coordinated, cell-type specific mechanisms.

## Results

### Diverse distribution of GlyRα1 subunits in spinal neurons

**Figure 1:**
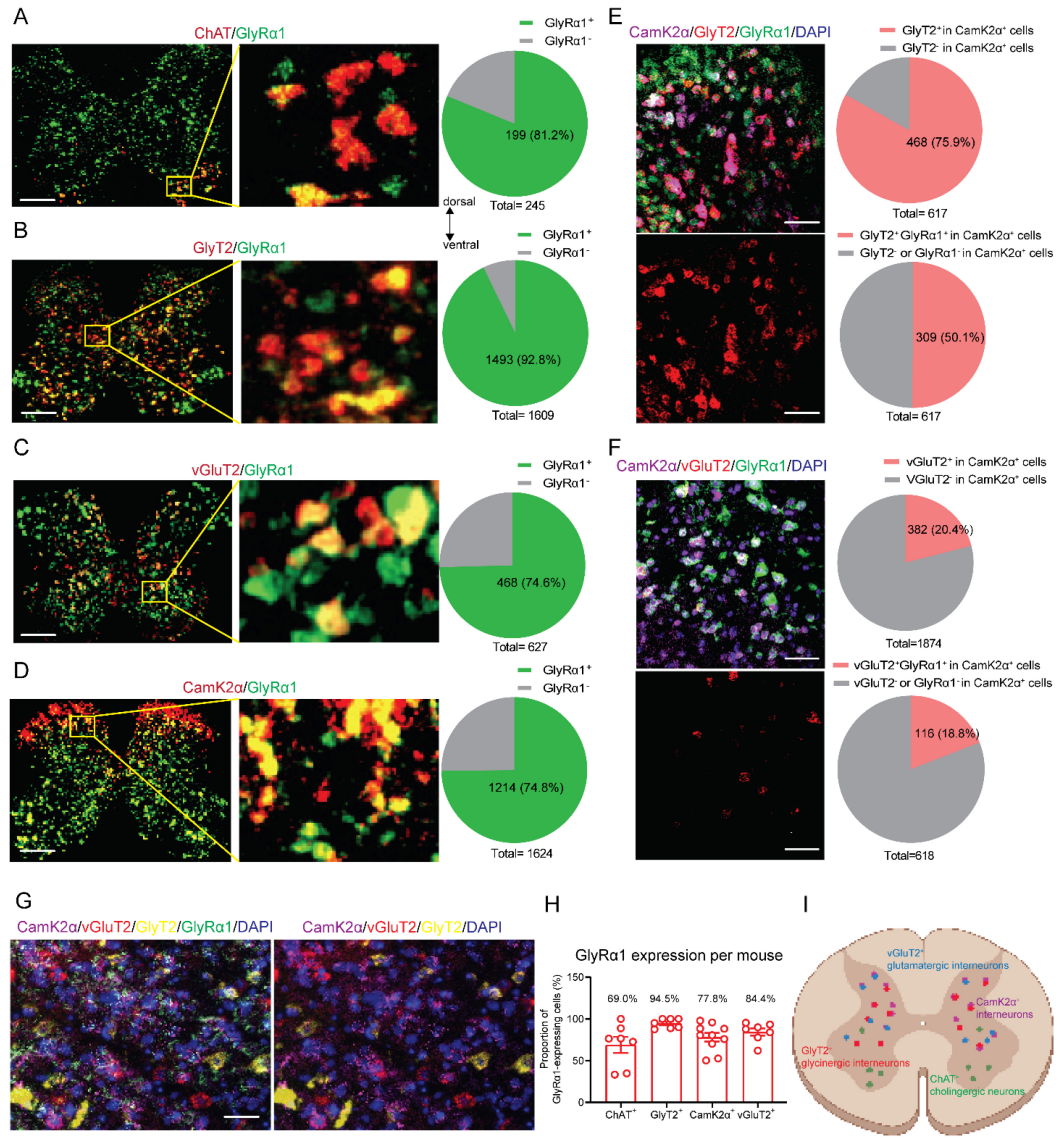
** Diverse distribution of GlyRα1 subunits in mouse spinal neurons. (A)** GlyRα1 mRNA expressed in 81.2% of ChAT-expressing neurons (a total of 245 neurons from 7 wildtype mice). **(B)** GlyRα1 mRNA expressed in 92.8% of GlyT2-expressing neurons (a total of 1609 neurons from 7 wildtype mice). **(C)** GlyRα1 mRNA expressed in 74.6% of vGluT2-expressing neurons (a total of 627 neurons from 7 wildtype mice). **(D)** GlyRα1 mRNA expressed in 74.8% of CamK2α-expressing neurons (a total of 1624 neurons from 10 wildtype mice).** (A-D)** Scale bar: 200 µm. **(E)** GlyT2 mRNA in 75.9% of CamK2α-expressing neurons, while GlyT2 and GlyRα1 mRNAs co-expressed in 50.1% of CamK2α-expressing cells in the spinal cord of wildtype mice (a total of 617 neurons from 4 wildtype mice). **(F)** vGluT2 mRNA in 20.4% of CamK2α-expressing neurons (a total of 1874 neurons from 10 wildtype mice), while vGluT2 and GlyRα1 mRNAs co-expressed in 18.8% of CamK2α-expressing cells in the spinal cord of wildtype mice (a total of 618 neurons from 4 mice). The images in **(E)** and **(F)** are from the dorsal horn area of spinal cord. Scale bar: 50 µm. **(G)** Labeling of the mRNAs of CamK2α, vGluT2, GlyT2 and GlyRα1 in the peri-canal region of the spinal cord using RNAscope Hiplex assay. The representative images are from the peri-central canal area of spinal cord. **(H)** GlyRα1 mRNA expressed in GlyT2-, ChAT-, CamK2α-, and vGluT2-expressing neurons averaged from each mouse (GlyT2: n = 7; ChAT: n = 7; CamK2α: n = 10; vGluT2: n = 7). **(I)** Schematic illustration of the relative expression of neuronal markers in mouse spinal cord. Error bars indicate mean±SEM.

We selected RNAscope to examine the cell-type distribution of GlyRα1 in spinal slices because this approach offers high sensitivity, specificity, and spatial resolution, enabling reliable detection of low-abundance transcripts. GlyRα1 mRNA labeling was detected in all neuronal subtypes examined, including cholinergic choline acetyltransferase (ChAT)-positive neurons (Figure 1A), glycine transporter type 2 (GlyT2)-positive neurons (Figure 1B), glutamatergic vesicular glutamate transporter 2 (vGluT2)-positive neurons (Figure 1C) and calcium/calmodulin-dependent protein kinase II alpha (CamK2α)-positive neurons (Figure 1D) in wildtype mice. The highest proportion of GlyRα1 mRNA signals was detected in glycinergic inhibitory interneurons (92.8%), for which GlyT2 serves as a biomarker.

CamK2α is expressed broadly in excitatory neurons in many brain regions 21. However, accumulating evidence suggests that CamK2α also colocalizes with glycinergic inhibitory neurons in the spinal cord 22, 23. We calculated the average cell-type specific expression of GlyRα1 across individual mice (n = 7-10). We observed that 75.9% of CamK2α signals co-localized with GlyT2-positive cells in the spinal cord, and 50.1% of spinal neurons exhibited co-localization of GlyT2, CamK2α and GlyRα1 mRNA labeling (Figure 1E). In parallel, 20.4% of CamK2α signals co-localized with vGluT2-positive cells in the spinal cord, and 18.8% of spinal neurons exhibited co-localization of vGluT2, CamK2α and GlyRα1 mRNA labeling in the spinal cord (Figure 1F). To further investigate the distribution of these neuronal markers, we used the RNAscope Hiplex assay to stain 5 subpopulation-specific biomarkers within a single tissue section (Figure 1G). This approach enables simultaneous detection of multiple RNA targets within the same tissue section with high sensitivity and spatial resolution. We found that there was no overlap between GlyT2 and vGluT2 in the spinal cord slice, while both neuronal populations exhibited a high proportion of CamK2α and GlyRα1 expression. The range of GlyRα1 co-localization with different neuronal biomarkers was 69.0%-94.5% in mouse spinal cord (Figures 1H and 1I). This suggests that CamK2α-positive neurons in the spinal cord predominantly include glycinergic inhibitory interneurons.

### Cell-type specific activation of GlyRα1-expressing neurons by startle stimulation

**Figure 2:**
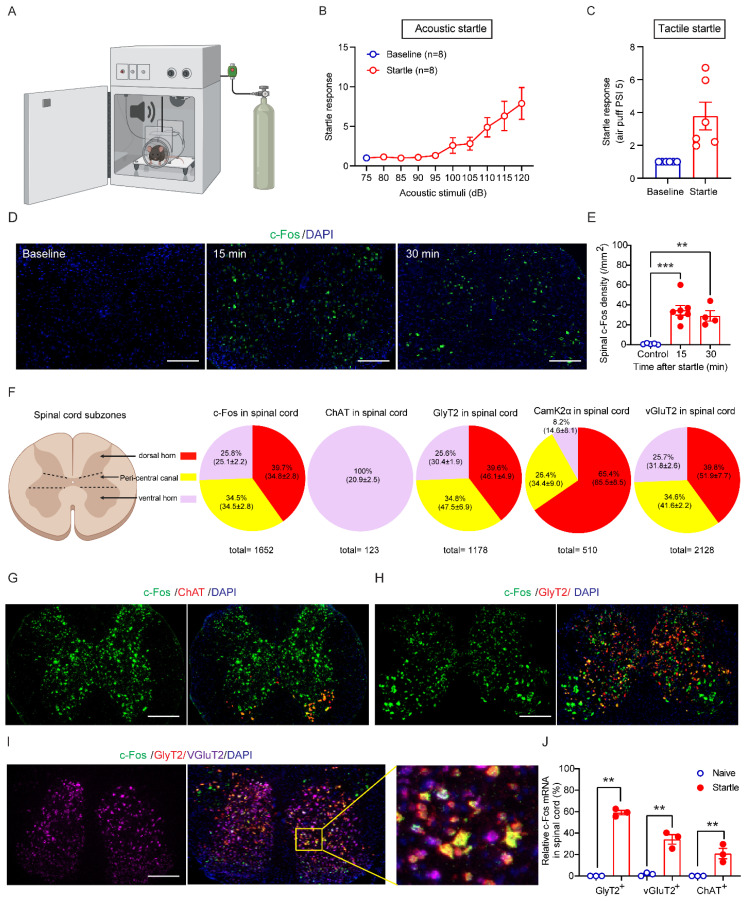
** Cell-type specific activation of GlyRα1-expressing neurons by startle stimulation. (A)** The startle box is designed for measuring startle responses induced by acoustic and tactile stimuli. **(B)** Startle responses induced by acoustic stimuli (n = 8 wildtype mice /group); **(C)** Startle responses induced by tactile stimuli (n = 6 wildtype mice /group); **(D-E)** Significantly increased densities of c-Fos mRNA in the spinal cord of wildtype mice, tissues collected 15 and 30 min after the startle response (Baseline: 0.8±0.3 /mm^2^, n = 5; 15 min: 34.5±4.8 /mm^2^, n = 7; 30 min: 29.1±5.2 /mm^2^, n = 4); **(F)** The expression percentage and densities of c-Fos and neuronal marker mRNAs in the spinal cord subzones of dorsal horn, peri-central canal area and ventral horn, respectively. For c-Fos mRNA quantification, a total of 1652 neurons from 7 wildtype mice were counted (dorsal horn vs peri-central canal vs ventral horn: ratio 39.7% vs 34.5% vs 25.8%; density, 34.8±2.8 vs 34.5±2.8 vs 25.1±2.2 /mm^2^); for ChAT mRNA quantification, 123 neurons from 4 wildtype mice were counted (dorsal horn vs peri-central canal vs ventral horn: ratio 0% vs 0% vs 100%; density, 0% vs 0% vs 20.9±2.5 /mm^2^); for GlyT2 mRNA quantification, 1178 neurons from 7 wildtype mice were counted (dorsal horn vs peri-central canal vs ventral horn: ratio 39.6% vs 34.8% vs 25.6%; density, 46.1±4.9 vs 47.5±6.9 vs 30.4±1.9 /mm^2^); for CamK2α mRNA quantification, 510 neurons from 7 wildtype mice were counted (dorsal horn vs peri-central canal vs ventral horn: ratio 65.4% vs 26.4% vs 8.2%; density, 65.5±8.5 vs 34.4±9.0 vs 14.6±8.1 /mm^2^); for vGluT2 mRNA quantification, 2128 neurons from 7 wildtype mice were counted (dorsal horn vs peri-central canal vs ventral horn: ratio 39.8% vs 34.6% vs 25.7%; density, 51.9±7.7 vs 41.6±2.2 vs 31.8±2.6 /mm^2^). **(G)** c-Fos expression in ChAT-expressing neurons; **(H)** c-Fos expression in GlyT2-expressing neurons; **(I)** c-Fos expression in GlyT2 and vGluT2-expressing neurons; **(J)** The density of c-Fos mRNA triggered by startle response in the spinal cords. The spinal cord tissues of wildtype mice were harvested 15 min after the startle stimulus and stained by RNAscope assay. Scale bar: 200 µm. One-way ANOVA with Tukey's multiple comparisons or unpaired *t* test were used, as appropriate. **p < 0.01; ***p < 0.001. Error bars indicate mean±SEM.

Startle responses to sound stimulation and air puff were dependent on the intensity of acoustic and tactile stimuli (Figures 2A, 2B and 2C). We next used RNAscope technique to examine startle-induced activation of c-Fos mRNA in different types of spinal neurons in wildtype mice. c-Fos activation is widely used to indicate engagement of specific neuronal populations by acoustic stimulation when combined with cell-type specific markers 24-26. In wildtype naive mice taken from their homecage, c-Fos mRNA signals were nearly undetectable in the spinal cord sections (control, Figure 2D). In contrast, acoustic stimuli induced robust spinal neuronal activation of c-Fos mRNA within 15 and 30 min (Figure 2E). c-Fos-expressing neuron density peaked at 15 min after the startle stimulus (Figure 2E).

Furthermore, we quantified the mRNA expression of c-Fos 15 min following the acoustic stimulation and spinal neuronal markers including ChAT, GlyT2, CamK2α and vGluT2 in the spinal cord subzones (Figure 2F). We found that 39.7% of c-Fos mRNA expressed in the dorsal horn, 34.5% of c-Fos mRNA in the peri-central canal area and 25.8% of c-Fos mRNA in the ventral horn, while 100% of ChAT mRNA expressed in the ventral horn.

Comparably, 39.6% of GlyT2 mRNA expressed in the dorsal horn, 34.8% of GlyT2 mRNA in the peri-central canal area and 25.6% of GlyT2 mRNA in the ventral horn, while 65.4% of CamK2α mRNA expressed in the dorsal horn, 26.4% of CamK2α mRNA in the peri-central canal area and 8.2% of CamK2α mRNA in the ventral horn. Regarding vGluT2 mRNA, 39.8% expressed in the dorsal horn, 34.6% in the peri-central canal area and 25.7% in the ventral horn. Next, we analyzed c-Fos expression in different neuronal subpopulations 15 min after acoustic startle stimulation. The c-Fos signals were detected in 58.3% of GlyT2-expressing neurons, 35.8% of vGluT2-expressing neurons and 20.9% of ChAT-expressing α-motor neurons, calculated based on the average of c-Fos densities of each mouse (Figure 2G-2J).

**Figure 3:**
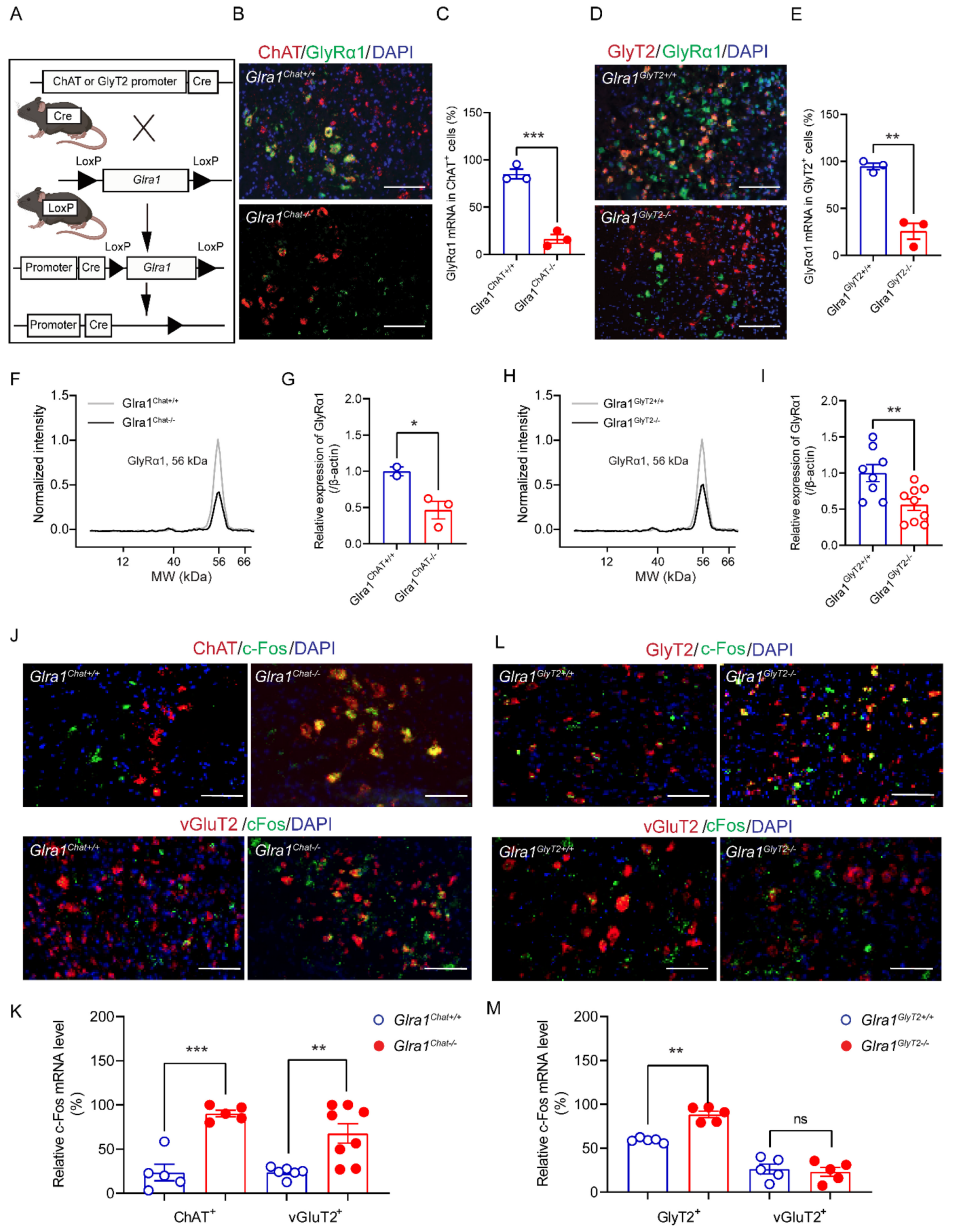
** GlyRα1 knockout affects startle-induced neuronal activity in the spinal cord. (A)** Schematic of generating the transgenic mice with Cre/LoxP system: *Glra1^Chat-/-^* and *Glra1^GlyT2-/-^* mice; **(B-C)** Significant reduction of GlyRα1 mRNA density was found in the spinal cord of *Glra1^Chat-/-^* mice, compared to the control (n = 3 /group). The representative images are from the ventral horn area of spinal cord. **(D-E)** Significant reduction of GlyRα1 mRNA density in the spinal cord of *Glra1^GlyT2-/-^* mice (n = 3 /group). The representative images are from the peri-central canal area of spinal cord. **(F-G)** Significant reduction of GlyRα1 protein expression in the spinal cord tissues of *Glra1^Chat-/-^* mice (n = 2-3 /group). **(H-I)** Significant reduction of GlyRα1 protein expression in the spinal cord tissues of *Glra1^GlyT2-/-^* mice (n = 8-9 /group). **(J-K)** The densities of c-Fos mRNA were significantly increased in both ChAT- and vGluT2-expressing neurons in the spinal cord of *Glra1^Chat-/-^* mice (n = 5-8 /group). The representative images are from (upper) the ventral horn and (lower) peri-central canal area of spinal cord. **(L-M)** The density of c-Fos mRNA was significantly increased in GlyT2-expressing, but not vGluT2-expressing neurons in the spinal cord of *Glra1^GlyT2-/-^* mice (n = 5 /group). The representative images are from the peri-central canal area of spinal cord. Scale bar: 50 µm. Unpaired *t* or Mann-Whitney test was used, as appropriate. *p < 0.05; **p < 0.01; ***p < 0.001; ns, not significant. Error bars indicate mean±SEM.

To explore the cell-type specific mechanisms underlying the startle response, we generated 2 conditional GlyRα1 deficient transgenic mouse lines by crossing *Glra1^flox/flox^* mice with the appropriate Cre-driver mouse lines, *GlyT2Cre* and *ChatCre*. The offspring of these transgenic mice included mice with deletion of GlyRα1 in the spinal α-motor neurons (*Glra1^Chat-/-^*) and mice with deletion of GlyRα1 in glycinergic neurons (*Glra1^GlyT2-/-^*) (Figure 3A). Homozygous *Glra1^Chat-/-^* mice exhibited disrupted co-localization of GlyRα1 with ChAT mRNA in the spinal cord sections, as revealed by RNAscope analysis (Figure 3B and 3C). Similarly, homozygous *Glra1^GlyT2-/-^* mice exhibited disrupted co-localization of *Glra1* with GlyT2 mRNAs in the spinal cord (Figure 3D and 3E). These transgenic mice also showed significantly reduced levels of GlyRα1 proteins in the lumbar tissues of spinal cord, measured with automated capillary-based electrophoresis Western blot assay (Figure 3F-3I). We next examined acoustic stimuli-induced c-Fos activation in spinal neurons from the transgenic mice. Deletion of GlyRα1 from ChAT-positive neurons selectively increased the number of c-Fos positive cells in ChAT- and vGluT2-expressing neurons (Figures 3J and 3K). In contrast, deletion of GlyRα1 from GlyT2-positive neurons significantly increased the density of c-Fos positive cells in GlyT2-positive neurons but not in ChAT- and vGluT2-positive neurons (Figure 3L and 3M). It suggests that activation of GlyRα1 subunits in the spinal cord normally suppresses neuronal activation, and that their deletion disinhibits the neuronal response to acoustic startle stimulation.

### GlyRα1 controls startle responses in a cell-type specific manner

**Figure 4:**
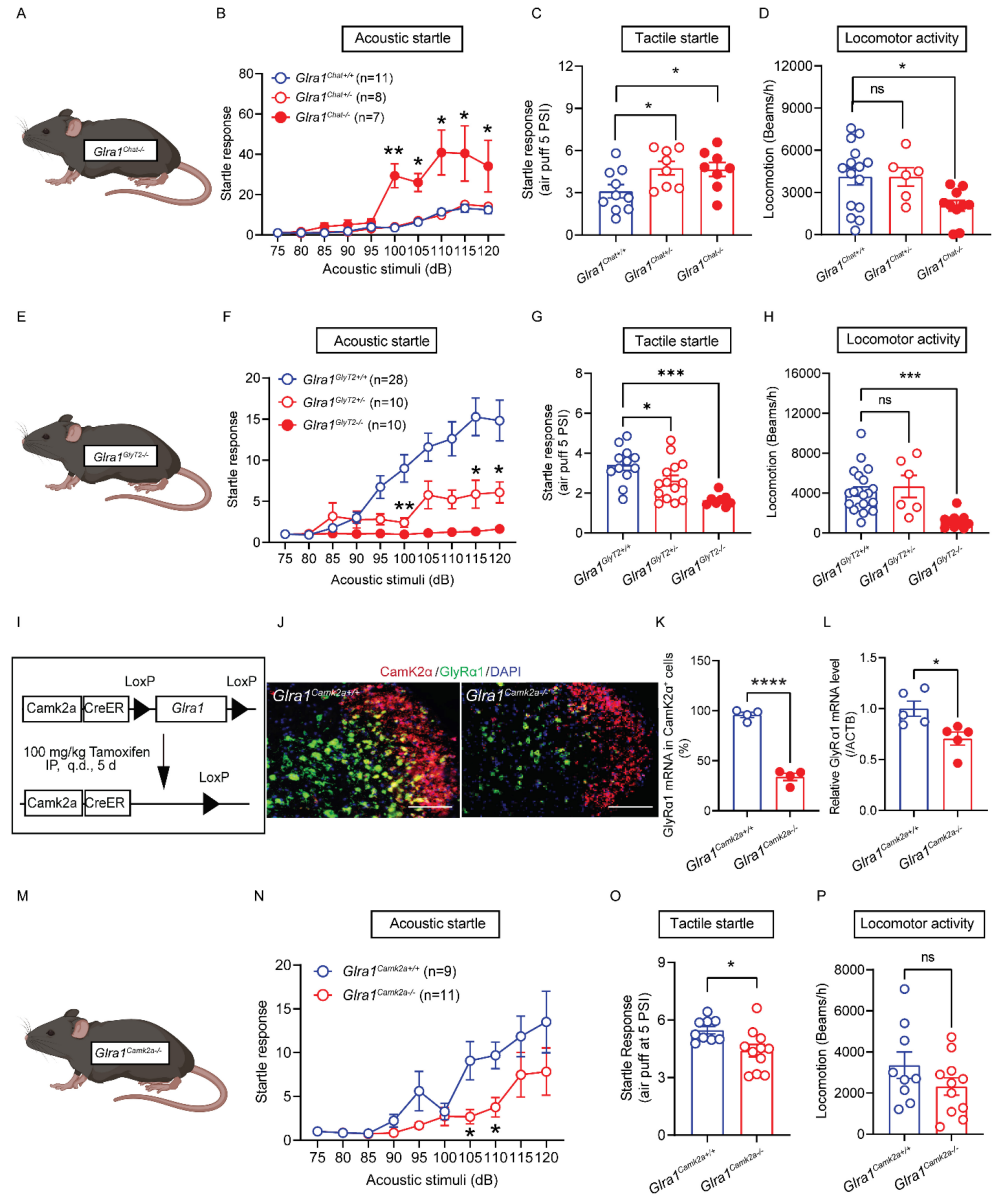
** GlyRα1 controls startle responses in a cell-type specific manner. (A-C)** *Glra1^Chat-/-^* mice showed a significantly enhanced acoustic startle response, compared to *Glra1^Chat+/-^* and* Glra1^Chat+/+^* mice, while *Glra1^Chat-/-^* and *Glra1^Chat+/-^* mice both exhibited a more pronounced tactile startle phenotype induced by air puff at 5 PSI. **(D)** However, *Glra1^Chat-/-^* mice showed decreased locomotor activity, compared to *Glra1^Chat+/+^* mice. **(E-F)** *Glra1^GlyT2+/-^* mice showed a significantly inhibited acoustic startle response, compared to* Glra1^GlyT2+/+^* mice, while there was nearly no acoustic startle response in *Glra1^GlyT2-/-^* mice. **(G)** Both *Glra1^GlyT2+/-^* and* Glra1^GlyT2-/-^* mice showed a significantly inhibited tactile startle response. **(H)** *Glra1^GlyT2+/-^* mice did not show changed locomotor activity, while *Glra1^GlyT2-/-^* mice exhibited significantly lower locomotor activity, compared to *Glra1^GlyT2+/+^* mice. **(I)** Schematic of an inducible *Glra1^Camk2a-/-^* mouse generation. **(J-K)** RNAscope staining confirmed that GlyRα1 mRNA level was significantly decreased in the spinal cord of *Glra1^Camk2a-/-^* mice. The representative images are from the dorsal horn area of spinal cord. **(L)** The reduction of GlyRα1 mRNA level in the spinal cord of *Glra1^Camk2a-/-^* mice was further verified by qPCR assay. **(M-O)** *Glra1^Camk2a-/-^* mice showed significantly decreased startle response, particularly at 105- and 110-dB acoustic stimuli, and suppressed tactile startle reflex, compared to control mice. **(P)** Conditional knockout of GlyRα1 from CamK2α-positive mice did not significantly alter their locomotor activity. Scale bar: 100 µm. One-way ANOVA with Tukey's multiple comparisons or unpaired *t* test were used, as appropriate. *p < 0.05; **p < 0.01; ***p < 0.001; ns, not significant. Error bars indicate mean±SEM.

We next investigated the *in vivo* consequences of the spinal cell-type specific activation elicited by startle stimulation observed in both ChAT- and GlyT2-GlyRα1 deficient mice. We first tested the sensitivity to acoustic stimuli of heterozygous and homozygous ChAT-GlyRα1 deficient mice (*Glra1^Chat+/-^* and *Glra1^Chat-/-^)* and compared them with their control littermates (*Glra1^Chat+/+^*, Figure 4A). Deletion of GlyRα1 subunits in the ChAT-positive neurons significantly increased the startle response to varying levels of acoustic stimulation in homozygous (*Glra1^Chat-/-^)*, but not heterozygous (*Glra1^Chat+/-^*), mice compared to the wildtype mice (Figure 4B). Unlike acoustic noise stimulation, heterozygous ChAT-GlyRα1 deficient mice also exhibited an enhanced tactile startle response compared with their wildtype littermates (Figure 4C). While homozygous ChAT-GlyRα1 deficient mice exhibited decreased locomotion, their heterozygous counterparts did not show a significant alteration in locomotion (Figure 4D). These observations suggested that GlyRα1 deficiency in spinal α-motor neurons resulted in an exaggerated startle response similar to the clinical manifestation observed in animals and humans with global GlyRα1 deficiency 3, 27. The homozygous transgenic mice did not show significant alterations in pain threshold, forelimb grip strength or body temperature (Supplementary Figure 1A-1D), suggesting that the alterations in startle response were not attributable to motor dysfunction or impaired nociception. We next examined the sensitivity to acoustic stimuli of mice with GlyRα1 deficiency in inhibitory GlyT2-positive neurons (Figure 4E). Deletion of GlyRα1 subunits in the GlyT2-positive neurons significantly decreased the startle response to varying levels of acoustic stimuli in both homozygous (*Glra1^GlyT2-/-^*) and heterozygous (*Glra1^GlyT2+/-^*) mice compared with the control littermates (*Glra1^GlyT2+/+^*, Figure 4F). Notably, heterozygous GlyT2-GlyRα1 deficient mice displayed reduced tactile startle response triggered by air puff at 5 PSI but not 1.5 PSI, compared to wildtypes (Figure 4G and Supplementary Figure 1E). Homozygous GlyT2-GlyRα1 deficient mice exhibited reduced locomotion, whereas their heterozygous counterparts did not show a significant alteration in locomotion (Figure 4H). Similar to the observations in ChAT-GlyRα1 deficient mice, GlyT2-GlyRα1 deficient mice did not exhibit altered pain threshold, forelimb grip strength or locomotor activity, suggesting that the *in vivo* impact of spinal GlyRα1-deficiency primarily affected the spinal cord motor function at the spinal lumbar level (Supplementary Figures 1F-1H).

GlyRα1 deficiency occurs throughout glycinergic inhibitory neurons in the spinal cord of *Glra1^GlyT2-/-^* mice, including thoracic segments that contain sympathetic preganglionic neurons. Thoracic spinal cord circuits are known to regulate sympathetic outflow to the heart. Therefore, loss of GlyRα1 in these circuits may alter autonomic control of cardiac function and the response to startle stimulation. In this regard, we assessed and compared metabolic and cardiovascular functions in *Glra1^GlyT2-/-^* mice and their wildtype littermates. We found that these transgenic mice did not show significant alterations in metabolic parameters including carbohydrate oxidation, respiratory quotient, fat oxidation, water or food consumption (Supplementary Figures 1I-1N). Furthermore, GlyRα1 deficiency restricted to GlyT2-expressing neurons did not significantly affect major load- and heart rate-dependent or -independent indices of left ventricular contractile function measured by pressure-volume approach (P-V; Supplementary Figures 2A-2B), including both systolic and diastolic parameters, compared with wildtypes (Supplementary Figures 2C-2Q). In addition, these mice also did not exhibit altered cross-sectional areas of cardiac muscle sections, compared to the wildtype (Supplementary Figure 2R). These findings suggest that spinal GlyRα1 subunits in the GlyT2-positive neurons play a role in the mediation of startle response, opposite to that of their counterparts in spinal α-motor neurons.

To further test this idea, we generated an inducible GlyRα1 deficient mouse line, *Glra1^Camk2a-/-^* mice (Figure 4I). In the spinal cord, GlyT2 signals are highly co-localized with CamK2α-positive cells, as shown in our data above. This inducible mouse model allows us to delete GlyRα1 receptor in the adult stage, thereby avoiding developmental confound. The co-localization of GlyRα1 mRNA with CamK2α signals was reduced by 66.1% in the spinal slices from *Glra1^Camk2a-/-^* mice, the tissues harvested 7-days after tamoxifen treatment of *Glra1^Camk2a-/-^* mice compared to the controls (Figure 4J and 4K). RT-qPCR analysis demonstrated the similar result showing that spinal GlyRα1 mRNA levels were decreased by 29.4% in *Glra1^Camk2a-/-^* mice (Figure 4L). Similar to the observation in GlyT2-GlyRα1 deficient mice, deletion of GlyRα1 subunit in CamK2α-positive neurons significantly reduced the startle response to varying levels of acoustic stimuli in mice (Figure 4M and 4N). CamK2α-GlyRα1 deficient mice also displayed a reduced startle response to air-puff-induced tactile startle response, compared to wildtypes (Figure 4O). CamK2α-GlyRα1 deficient mice did not show significant alterations in locomotor activity, motor coordination in the rotarod test, body temperature or pain sensitivity in the tail flick test (Figure 4P, Supplementary Figures 3A-3C). Collectively, these observations suggest that GlyRα1 deficiency in the spinal inhibitory interneurons suppresses startle response, in contrast to the effect of GlyRα1 deficiency in spinal α-motor neurons.

### Cell-type specific regulation of startle responses by spinal GlyRα1 subunits

**Figure 5:**
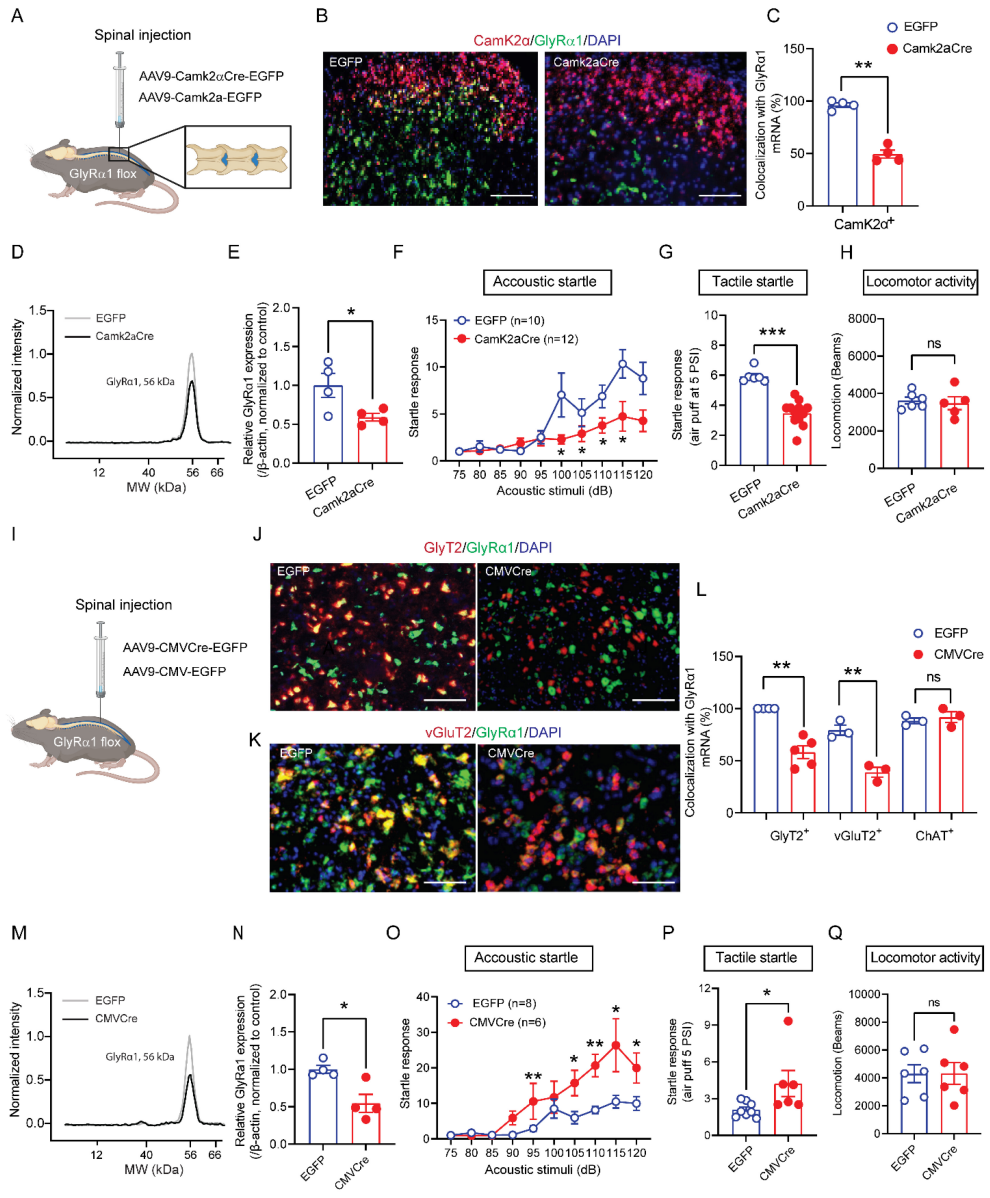
** Cell-type specific regulation of startle responses by spinal GlyRα1 subunits. (A)** Schematic of spinal injections of *AAV9-Camk2aCre-EGFP* and the control* AAV9-Camk2a-EGFP*. **(B-C)** Spinal injections of *AAV9-Camk2aCre-EGFP* significantly decreased the level of GlyRα1 mRNA by nearly 50% in CamK2α-positive neurons, verified with RNAscope assay. The representative images are from the dorsal horn of the spinal cord. Scale bar: 100 µm. **(D-E)** Expression of GlyRα1 protein in the spinal cord was remarkably reduced by *AAV9-Camk2aCre-EGFP.* **(F-G)** Spinal injections of *AAV9-Camk2aCre-EGFP* inhibited startle responses induced by acoustic stimuli and tactile air puff, respectively. **(H)** Spinal injections of *AAV9-Camk2aCre-EGFP* did not affect locomotor activity. **(I)** Schematic of spinal injections of *AAV9-CMVCre-EGFP* and the control *AAV9-CMV-EGFP*. **(J-L)** Spinal injections of *AAV9-CMVCre-EGFP* significantly decreased the levels of GlyRα1 mRNA in GlyT2- and vGluT2-expressing neurons, but not in ChAT-expressing neurons in the spinal cord. The representative images are from the peri-central canal area of spinal cord. Scale bar: **(J)** 50 µm and **(K)** 30 µm. **(M-N)** Expression of GlyRα1 protein in the spinal cord tissues was remarkably reduced by spinal injection of *AAV9-CMVCre-EGFP.* **(O-P)** Spinal injections of *AAV9-CMVCre-EGFP* significantly enhanced startle responses induced by acoustic stimuli and air puff, respectively. **(Q)** Spinal injection of *AAV9-CMVCre-EGFP* did not significantly affect locomotor activity. One-way ANOVA with Tukey's multiple comparisons or unpaired *t* test were used, as appropriate. *p < 0.05; **p < 0.01; ***p < 0.001; ****p < 0.0001; ns, not significant. Error bars indicate mean±SEM.

Depletion of GlyRα1 subunits in CamK2α-expressing neurons resulted in decreased startle responses, as shown above. This raises a question of whether spinal GlyRα1 subunits regulate startle response through a cell-type specific mechanism. To test this hypothesis, we delivered *AAV9-Camk2aCre-EGFP* into the lumbar spinal cord of GlyRα1 flox mice*,* respectively (Figure 5A). Intraspinal deletion of GlyRα1 from CamK2α-positive neurons significantly reduced the co-localization of GlyRα1 with CamK2α mRNA signals (Figure 5B and 5C), as well as the levels of GlyRα1 protein expression (Figure 5D and 5E). Deletion of spinal GlyRα1 from CamK2α-positive neurons reduced startle response induced by both acoustic and tactile stimulation (Figures 5F and 5G). This spinal GlyRα1 deletion induced by *AAV9-Camk2aCre-EGFP* injection did not affect locomotor activity in mice (Figures 5H). Inhibition of the startle reflex by intraspinal deletion of GlyRα1 subunits in CamK2α-positive neurons is consistent with the *in vivo* phenotype of CamK2α-GlyRα1 deficient mice as well as GlyT2-GlyRα1 deficient mice. This contrasts with the phenotype in the mice with global GlyRα1 deficiency, which consistently demonstrates hyperekplexia-like behavior. However, whether spinal deletion of GlyRα1 subunits can reproduce a similar phenotype has not been reported. To address this question, we injected *AAV9-CMVCre-EGFP* into the spinal cord of GlyRα1 flox mice (Figures 5I). We first used RNAscope assay to examine the cell-type specific co-localization of GlyRα1 mRNA signals in spinal slices. Intraspinal injection of *AAV9-CMVCre-EGFP* significantly reduced GlyRα1 co-localization with GlyT2 and vGluT2 mRNA signals without significantly altering GlyRα1 co-localization with ChAT signals (Figure 5J-5L). The expression levels of GlyRα1 protein were also reduced in the spinal cord tissues of the mice with intraspinal *AAV9-CMVCre-EGFP* injection (Figure 5M and 5N). These animals with spinal deletion of GlyRα1 exhibited pronounced startle reflexes in response to both acoustic and tactile stimuli (Figure 5O and 5P), recapitulating the phenotype previously described in global GlyRα1 deficient mice 3. Meanwhile, this GlyRα1 deletion induced by spinal virus injection did not affect locomotor activity in the mice (Figure 5Q).

Intraspinal injection of *AAV9-CMVCre-EGFP* in GlyRα1 flox mice did not produce a detectable EGFP signal in the brainstem slices, indicating that expression was limited to the spinal cord (Supplementary Figure 4). Together, these findings suggest that spinal GlyRα1 subunits regulate startle response in a cell-type specific manner.

### Deletion of GlyRα1 subunits in CamK2α-positive cells from brainstem RtTg increases startle responses

**Figure 6:**
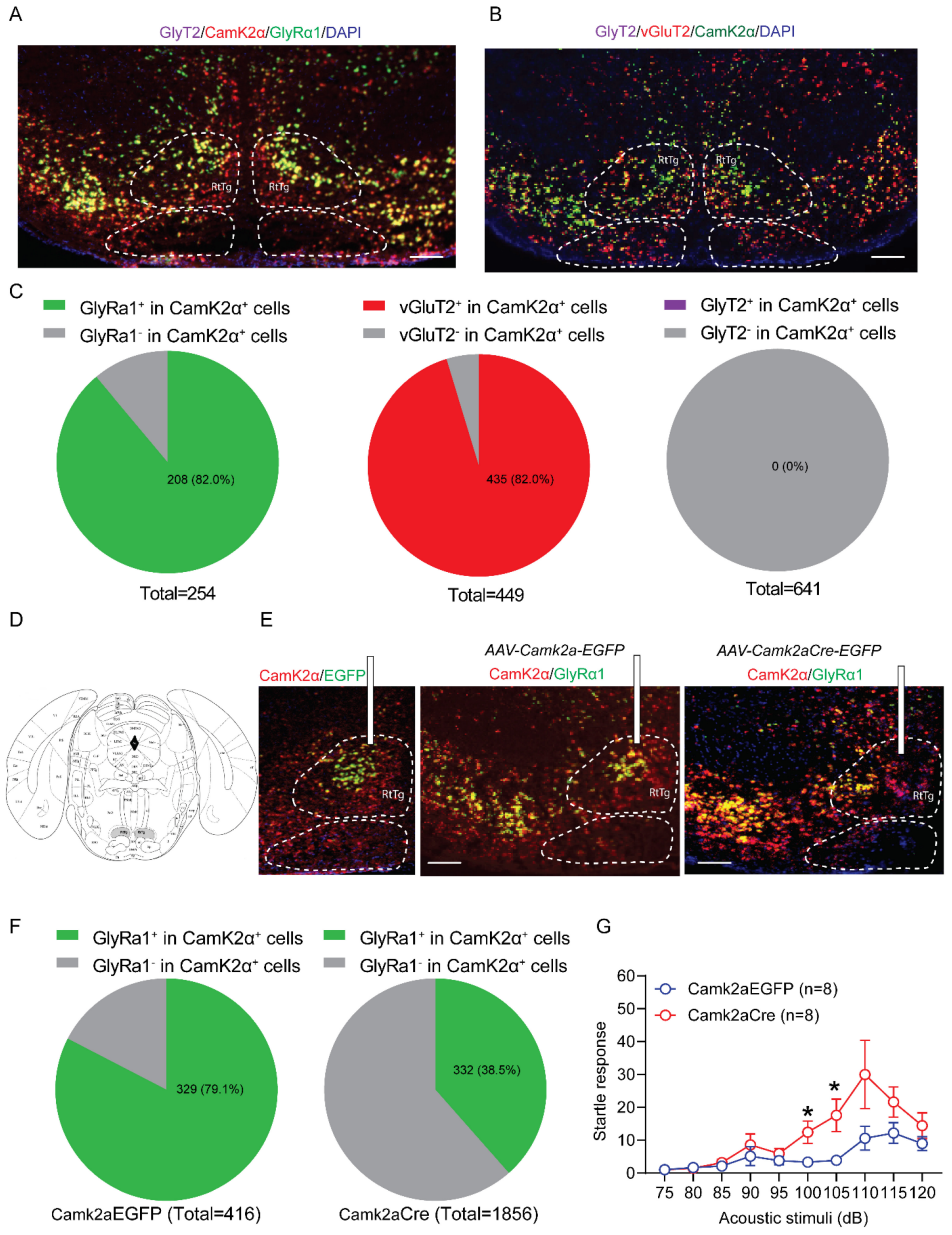
** Deletion of GlyRα1 in CamK2**α**-positive neurons from brainstem RtTg increases startle responses. (A-B)** Representative images for the mRNAs of CamK2α, vGluT2, GlyRα1 and GlyT2 in the RtTg. **(C)** Quantification of GlyRα1, vGluT2 and GlyT2 mRNA levels in CamK2α-positive neurons (GlyRα1 vs vGluT2 vs GlyT2: 82.0% vs 82.0% vs 0%) in brainstem RtTg. Neuron counts in the RtTg slices were derived from 4 mice. **(D)** Diagram and localization of the RtTg in brainstem (in grey). **(E)** Schematic of stereotaxic injections of *AAV9-Camk2aCre-EGFP* and the control *AAV9-Camk2a-EGFP*. **(F)** Injections of *Camk2aCre-EGFP* significantly decreased the level of GlyRα1 mRNA in CamK2α-positive neurons in the RtTg, from 79.1% in control group (a total of 416 neurons from 4 mice) to 38.5% in Camk2aCre-injecting group. Scale bar: 150 µm. (**G**) Specific knockout of GlyRα1 in CamK2α-positive neurons from brainstem RtTg increased acoustic startle responses. One-way ANOVA with Tukey's multiple comparisons or unpaired t test were used, as appropriate. *p < 0.05; ns, not significant. Error bars indicate mean±SEM.

Both *Glra1^Camk2a-/-^* mice and spinal deletion of GlyRα1 in CamK2α-positive cells reduced the acoustic startle response, as shown above. This suggests that CamK2α-driven GlyRα1 deletion predominantly affects glycinergic inhibitory interneurons at the spinal cord level. In contrast, CamK2α is widely expressed in excitatory neurons at supraspinal levels 28, 29. In view of this, we examined the role of GlyRα1 in brainstem CamK2α-positive cells in modulating acoustic startle response, with a particular focus on the RtTg, a key component of the circuitry that transmits startle signals from sensory input to motor output. No detectable GlyT2-positive signal was observed in this region, and CamK2α expression was predominantly colocalized with excitatory vGluT2-expressing neurons (Figure 6A and 6B). Notably, 82.0% of CamK2α-positive neurons expressed GlyRα1, matching the proportion that expressed vGluT2 (82.0%). No GlyT2 mRNA signals were found in the RtTg (Figure 6C). To explore the role of GlyRα1 in supraspinal CamK2α-positive neurons, we selectively deleted GlyRα1 in the RtTg by injecting *AAV9-CamK2α-Cre* into *Glra1* floxed mice (Figure 6D). AAV-selective deletion of CamK2α-specific GlyRα1 in RtTg significantly reduced the colocalization of GlyRα1 with CamK2α in the RtTg (Figure 6E and 6F). Deletion of GlyRα1 from RtTg CamK2α-positive neurons exhibited an increased startle response to acoustic stimulation (Figure 6G), in contrast to the reduced response observed in mice with GlyRα1 deletion in spinal CamK2α neurons.

### RNA-seq analysis: GlyT2-GlyRα1 deficiency promotes the changes in gene expression profiles in spinal cord

**Figure 7:**
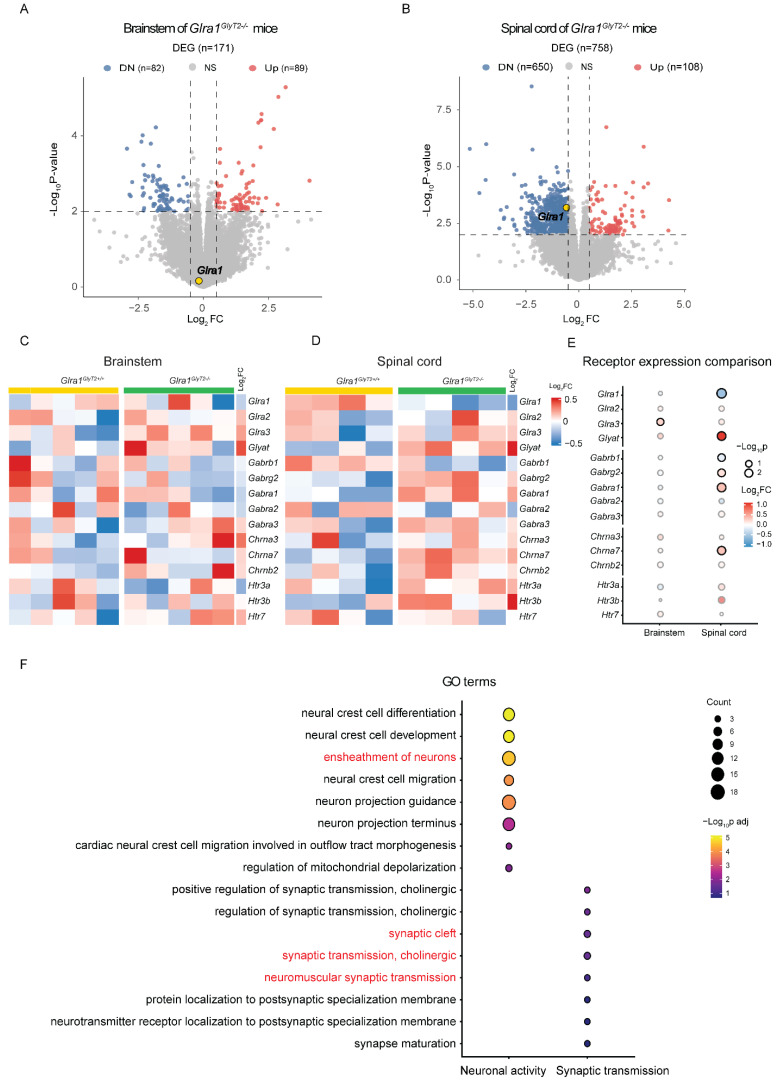
** RNA-seq analysis: GlyT2-GlyRα1 deficiency promotes the changes in gene expression profiles in spinal cord. (A)** Volcano maps showed that the levels of GlyRα1 mRNA did not differ in the brainstem of *Glra1^GlyT2-/-^* mice (n = 5 /group), while **(B)** the level was significantly decreased in spinal cord of *Glra1^GlyT2-/-^* mice (n = 4 /group), compared to the controls. **(C-D)** Heatmaps of gene expression showed GlyRα1 mRNA level in the spinal cord rather than in the brainstem was significantly reduced, based on the RNA-seq data from the tissues of *Glra1^GlyT2+/+^* and *Glra1^GlyT2-/-^* mice. Collectively, there were more differentially expressed genes (DEGs) in the spinal cord (n = 758) than in the brainstem (n = 171) of *Glra1^GlyT2-/-^* mice, suggesting a dominant change probably happened in the spinal cord rather than the brainstem of *Glra1^GlyT2-/-^* mice. **(E)** The mRNA levels of ligand-gated ion channel receptors (LGICs) in the brainstem and spinal cord were compared between *Glra1^GlyT2-/-^* and *Glra1^GlyT2+/+^* mice, respectively, suggesting that spinal cord showed a more significant change in the level of GlyRα1 mRNA, compared to that in brainstem. **(F)** Gene ontology (GO) showed that the terms related to “*neuronal activity*” and “*synaptic transmission*” were significantly enriched in the spinal cord of *Glra1^GlyT2-/-^* mice.

We conducted RNA sequencing to examine the molecular impact of GlyT2-GlyRα1 deficiency in the brainstem and spinal cord. The tissues were collected from GlyT2-GlyRα1 deficient mice and their control littermates. GlyRα1 subunits belong to a superfamily of Cys-loop ligand-gated ion channels (LGICs), which includes GABA_A_ receptors, nicotinic acetylcholine receptors (nAChRs), and 5-HT_3_ receptors 27, 30, 31. These ion channel receptors share a degree of structural and functional similarity. In this regard, we next asked whether the decreased GlyRα1 subunit expression leads to the alterations in the expression levels of other LGIC members. Thus, we analyzed the gene expression profiles of the members of the cys-loop ligand-gated ion channel superfamily, to which GlyRα1 subunits belong. The volcano plots indicated that the spinal cord exhibited more pronounced and dynamic transcriptional changes than the brainstem, when comparing the transgenic mice with their own control (Figure 7A and 7B). For instance, 758 differentially expressed genes (DEGs) were identified in the spinal cord, compared with only 171 DEGs in the brainstem, suggesting a higher relevance of spinal cord than brainstem in the involvement of the changed startle response in GlyT2-GlyRα1 deficient mice. A parallel comparison of brainstem and spinal cord heatmaps revealed differential expression of GlyRα1 subunits in the spinal cord but not in the brainstem tissues, when comparing GlyT2-GlyRα1 deficient mice with their normal controls (Figure 7C and 7D). By calculating the ratio of target gene expression levels in the spinal cord to that in the brainstem, we observed more significant alterations of the gene expression in the spinal cord than those in the brainstem (Figure 7E). To further explore the biological insight of GlyT2-GlyRα1 deficiency-induced DEGs, we used functional enrichment analysis to identify the possible signaling pathways involving in these profile changes (Figure 7F), including the terms “*neuromuscular synaptic transmission*”, “*synaptic cleft*”, “*cholinergic synaptic transmission*”, “*ensheathment of neurons*” and so on, indicating a close relationship between synaptic transmission and GlyRα1 expression in the spinal cord tissues but not in the brainstem tissues (Figure 7F).

Furthermore, we conducted RNA sequencing using the brainstem and spinal cord tissues from ChAT-GlyRα1 deficient mice and the control littermates. We found that the levels of GlyRα1 mRNA did not differ in the brainstem of *Glra1^Chat-/-^* mice, while the level was significantly decreased in the spinal cord of *Glra1^Chat-/-^* mice, compared to the controls (Supplementary Figure 5A and 5B). GlyRα1 mRNA level in the spinal cord rather than in the brainstem was significantly reduced (Supplementary Figure 5C, 5D and 5E). Gene ontology (GO) showed that the GO terms mainly related to *neuronal activity* were significantly enriched in the spinal cord of *Glra1^Chat-/-^* mice (Supplementary Figure 5F).

### GlyRα1 deficiency in GlyT2-expressing neurons reduces glycine currents

**Figure 8:**
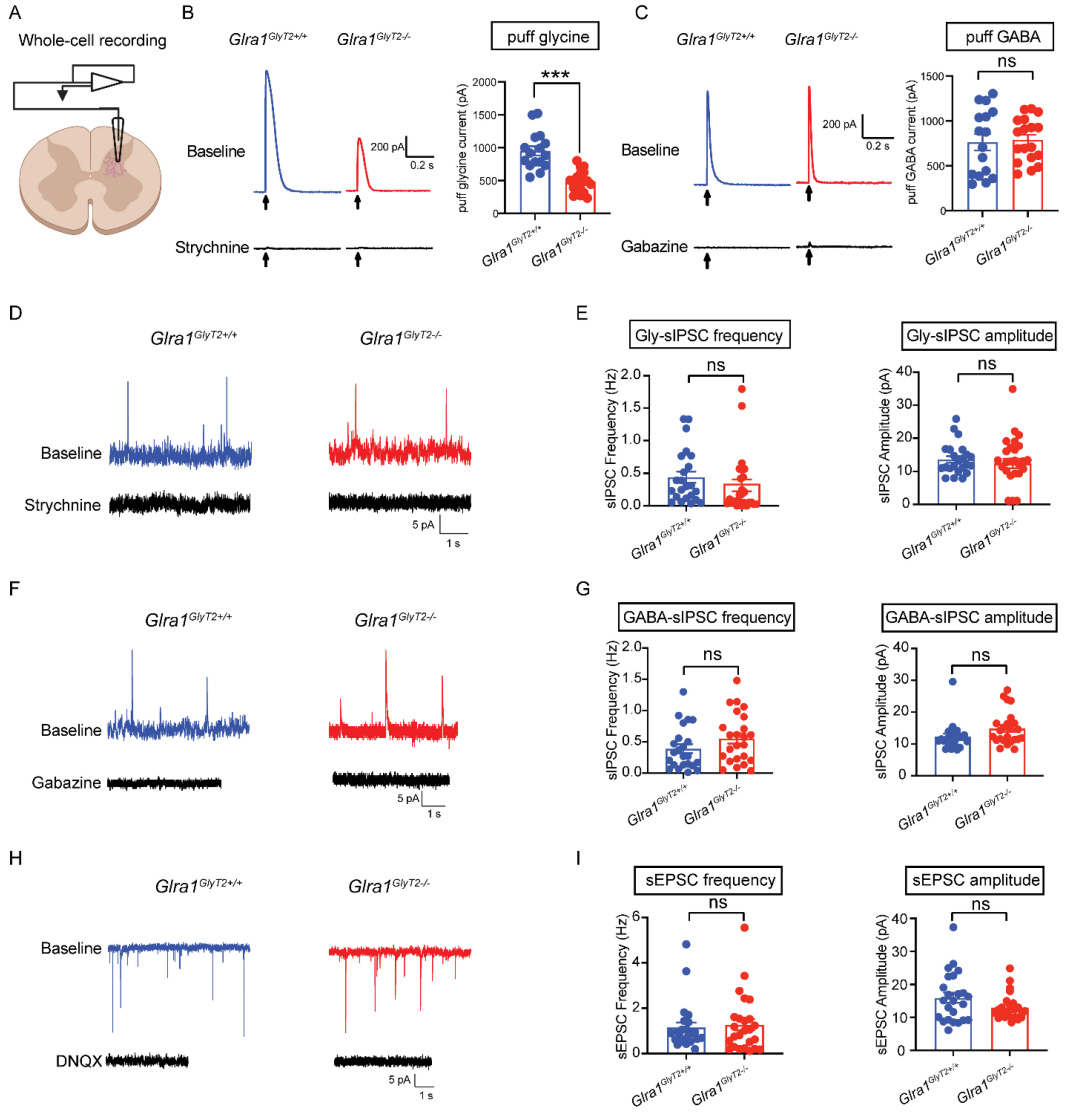
** GlyRα1 deficiency in GlyT2-expressing neurons reduces glycine currents. (A)** Schematic of electrophysiological whole-cell recording on the spinal interneurons of *GlyT2Cre;Glra1^flox/flox^-Tdtomato* mice. **(B-C)** Conditional deletion of GlyRα1 from GlyT2-expressing neurons significantly reduced the amplitude of puff-applied glycine-evoked currents (*Glra1^GlyT2+/+^*, n = 16 neurons; *Glra1^GlyT2-/-^*, n = 18 neurons), without affecting the currents elicited by puff-applied GABA (*Glra1^GlyT2+/+^*, n = 16 neurons; *Glra1^GlyT2-/-^*, n = 18 neurons), in the spinal cord slices. **(D-E)** Conditional deletion of GlyRα1 from GlyT2-expressing interneurons did not affect the frequency or amplitude of glycinergic spontaneous inhibitory postsynaptic currents (sIPSCs) in the spinal cord (*Glra1^GlyT2+/+^*, n = 23 neurons; *Glra1^GlyT2-/-^*, n = 25 neurons). **(F-G)** Conditional deletion of GlyRα1 from GlyT2-expressing neurons did not affect the frequency or amplitude of GABAergic sIPSCs in the spinal cord (*Glra1^GlyT2+/+^*, n = 23 neurons; *Glra1^GlyT2-/-^*, n = 24 neurons). **(H-I)** Conditional deletion of GlyRα1 from GlyT2-expressing neurons did not affect the frequency or amplitude of spontaneous excitatory postsynaptic currents (sEPSCs) in the spinal cord (*Glra1^GlyT2+/+^*, n = 24 neurons; *Glra1^GlyT2-/-^*, n = 25 neurons). In this test, we used strychnine as a specific competitive antagonist for inhibitory glycine receptors, gabazine as a selective antagonist for GABA_A_ receptors, and DNQX or 6,7-dinitroquinoxaline-2,3-dione as a selective competitive antagonist for non-NMDA glutamate receptors. Here, 1 mM glycine, 2 µM strychnine ,100 μM GABA and 10 µM gabazine, and 10 µM DNQX were applied in this test. Unpaired *t* test was used to compare the difference between 2 groups. ***p < 0.001; ns, not significant. Error bars indicate mean±SEM.

Structural or functional disruption of GlyRα1 subunits often diminishes glycinergic inhibitory neurotransmission and can also produce secondary alterations in GABAergic transmission in rodent models 32-34. We next asked whether selective deletion of GlyRα1 subunits from GlyT2-expressing neurons affects either glycinergic or GABAergic inhibitory postsynaptic currents (IPSCs). To address this, we performed whole-cell patch-clamp recordings on the spinal cord slices from GlyT2Cre-tdTomato-tagged mice, which enabled the identification and targeted recording of GlyT2-expressing neurons (Figure 8A). We observed that GlyT2-GlyRα1 subunit deficiency substantially reduced the amplitude of currents evoked by local application of glycine (Gly), but not those activated by GABA application, compared with the wildtypes (Figures 8B and 8C). We next examined whether GlyT2-GlyRα1 deficiency affects glycinergic and GABAergic spontaneous IPSCs (sIPSCs). Depletion of GlyRα1 subunits in GlyT2-expressing neurons did not significantly alter the frequency or amplitude of either glycinergic or GABAergic sIPSCs in the spinal cord slices (Figures 8D-8G). Similarly, knockout of GlyRα1 subunits from GlyT2-expressing neurons also did not significantly affect the frequency or amplitude of spontaneous excitatory postsynaptic currents (sEPSCs) in the spinal cord slices (Figures 8H-8I). Together, these findings suggest that GlyT2-targeted GlyRα1 deletion reduces non-synaptically evoked glycine receptor currents without measurably altering spontaneous synaptic transmission under our recording conditions, raising the possibility that extrasynaptic GlyRα1 receptors contribute to startle regulation.

## Discussion

The data presented in this study suggest that the cell-type specific distribution of GlyRα1 subunits is essential for balancing the startle response through coordinated fast excitatory signaling with the precise inhibitory control. This conclusion is supported by several lines of evidence. First, selective deletion of GlyRα1 subunits from excitatory or inhibitory neurons differentially disinhibited startle-induced c-Fos activation. Second, selective deletion of GlyRα1 subunits from GlyT2 interneurons suppressed the startle response, whereas deletion of GlyRα1 subunits from motor neurons increased acoustic startle response. Third, this opposing regulation by GlyRα1 across neuronal populations was recapitulated when GlyRα1 subunits were selectively deleted in the spinal cord. Specifically, GlyRα1 deficiency in CamK2α-positive cells produced region-dependent effects on the startle response. At the spinal level, CamK2α-driven GlyRα1 deficiency primarily disinhibited GlyT2-positive inhibitory interneurons, thereby suppressing the startle response. In contrast, at the brainstem level, CamK2α-driven GlyRα1 deficiency predominantly occurred in excitatory glutamatergic neurons, thereby enhancing excitatory neurotransmission and startle response. Finally, GlyT2- or ChAT-GlyRα1 deficiency appeared to have a larger impact on spinal cord than brainstem, as revealed by RNA-seq analysis. For example, GlyRα1 deficiency was observed in the spinal cord tissues but not in the brainstem. Moreover, deletion of GlyRα1 from GlyT2 neurons altered gene expression profiles, and such alteration was more pronounced in the spinal cord than the brainstem. Collectively, these findings provide evidence for a cell-type specific mechanism in the spinal cord by which GlyRα1 subunits critically regulate startle responses induced by both acoustic and tactile stimulations in mice.

GlyT2 is a plasma membrane glycine transporter, responsible for glycine uptake at presynaptic terminals 35. GlyT2-expressing neurons are most abundantly found in the spinal cord and brainstem, but are largely undetectable in higher brain regions 36. In the spinal cord, GlyT2 neurons are exclusively inhibitory and glycinergic. Similar to point mutations in the GlyRα1 subunit, point mutations in GlyT2 also result in glycinergic deficiency and hyperekplexia, characterized by exaggerated startle responses in rodents 3, 37. However, one interesting finding from our study is that GlyRα1 depletion from GlyT2-expressing neurons reduced acoustic and tactile stimuli-induced startle responses. This startle-suppressing effect is likely due to disinhibition of GlyT2-positive inhibitory interneurons, thereby enhancing inhibition of glutamatergic interneurons or α-motor neurons. This idea is further supported by our c-Fos experiments in spinal cord slices from cell-type specific GlyRα1 deficient transgenic mice. c-Fos activity was selectively increased in GlyT2-expressing neurons, but not in vGluT2- or ChAT-expressing neurons, in GlyT2-GlyRα1 deficient mice. CamK2α-GlyRα1 deficient mice exhibited a suppression of startle responses, similar to that seen in GlyT2-GlyRα1 deficient mice. In contrast, selective deletion of GlyRα1 subunits from ChAT-expressing neurons significantly enhanced c-Fos activity in both vGluT2- and ChAT-expressing neurons, but not GlyT2-expressing neurons. Thus, the pattern of c-Fos expression matched well with startle responses from several cell-type specific GlyRα1 deficient mice. Collectively, our findings suggest that both excitatory and inhibitory neurons contribute to startle reflexes, and their activity levels are fine-tuned by GlyRα1 subunits.

The brainstem has been identified as the center for the initiation of startle response 1-3, 38. The spinal cord is thought to simply relay the commands from the brainstem and to coordinate muscle contraction via motor neuron subtypes.

The suppressive effect induced by intraspinal deletion of spinal GlyRα1 subunits in CamK2α-expressing neurons is consistent with the startle phenotype in CamK2α-GlyRα1 deficient mice. In the spinal cord, CamK2α primarily labeled glycinergic inhibitory neurons, as revealed by our RNAscope analysis. This is not the case in the brainstem, where CamK2α is considered a marker of excitatory neurons because it is highly co-localized with vGluT2 mRNA. In contrast to the reduced response observed in mice with GlyRα1 deletion in spinal CamK2α-positive neurons, RtTg CamK2α-neurons specific GlyRα1-deficient mice exhibited an increased startle response to acoustic stimulation. This observation explains the distinct role of GlyRα1 subunits in GlyT2/CamK2α-positive neurons in spinal cord in regulation of startle phenotype. Our finding is in line with a previous report that depletion of glutamatergic neurons in the brainstem RtTg leads to a significant reduction of startle behavior 1, 2. In contrast to the well-studied brainstem circuits, spinal cord mechanisms controlling the startle response are less well defined. Here, we provide evidence for a cell-type specific mechanism by which spinal GlyRα1 subunits regulate startle responses induced by both acoustic and tactile stimulations in mice. GlyRα1 subunits were broadly expressed in α-motor neurons and excitatory vGluT2-expressing neurons, and were most abundant in GlyT2-expressing inhibitory interneurons in the spinal cord. This cell-type specific distribution of GlyRα1 subunits is essential for balancing the startle response through coordinated fast excitatory signaling with the precise inhibitory control. Startle-induced c-Fos activation of CamK2α neurons was predominantly observed in the spinal dorsal horn, a region enriched in sensory neurons, suggesting that local sensory inputs within the lumbar spinal cord play a role in shaping the execution of startle behaviors. This idea is consistent with previous studies that local sensory inputs in the spinal cord influence inhibitory interneuron activity and the selection of startle behaviors in zebrafish 39, 40, as well as the findings that spinal cord injury in humans alters the latency and amplitude of startle reflexes to somatosensory stimuli 41.

Previous studies from our laboratory and others found that dysfunction of GlyRα1 receptors leads to impairments in both presynaptic and postsynaptic glycinergic and GABAergic neurotransmission in rodents 3, 34, 42, 43. However, depletion of GlyRα1 subunits from GlyT2-expressing neurons did not significantly alter either glycinergic or GABAergic sIPSCs. Instead, we observed a reduction in puff Gly-evoked currents in GlyT2-positive neurons, suggesting that extrasynaptic GlyRα1 receptors may contribute to spinal mechanisms regulating startle reflexes. Extrasynaptic GlyRs primarily mediate a continuous “tonic” inhibition that sets overall neuronal excitability, contrasting with synaptic GlyRs that handle fast “phasic” signals. Extrasynaptic GlyRs, particularly GlyRα1 subunits, play an important modulatory role in the startle response 44, 45. Dysfunction of extrasynaptic GlyRs can lead to hyperactive startle responses, as observed in hyperekplexia models 32. Most evidence for extrasynaptic GlyRs comes from the studies in the brainstem, with limited data from spinal cord experiments. Unfortunately, we did not detect GlyR-mediated tonic currents in spinal cord slices. This limits further exploration of extrasynaptic GlyRs in cell-type specific regulation of the startle response. Notably, both homozygous GlyT2-GlyRα1 and ChAT-GlyRα1 knockout mice exhibited reduced locomotor activity, whereas their heterozygous counterparts showed no significant alterations in general neurological behaviors, including locomotion. Interestingly, these heterozygous mice, similar to their homozygous littermates, displayed the opposite effects on startle behaviors. These findings suggest that the effects of GlyRα1 deficiency in distinct cell-types on startle responses are independent of the motor impairment observed in homozygous GlyRα1 deficient mice.

Collectively, we identify a spinal mechanism in which the coordinated actions of excitatory and inhibitory neurons shape startle reflexes. GlyRα1 subunits in the spinal cord play a central role in this regulatory pathway. Expressed in distinct neuronal populations, these receptors in the spinal cord represent previously underappreciated targets for modulating motor and sensory functions and for understanding related neurological disorders.

## Material and Methods

### Mice

All male mice used in this study were on a C57BL/6J background and housed in a pathogen-free facility with constant temperature (23±1 ℃) and humidity (50±10%) on a 12h/12h light/dark cycle (lights on at 06:30 am and off at 06:30 pm). Behavioral tests were carried out in a double-blind manner. *Camk2aCreERT2* (Cat#012362), *GlyT2Cre* (Cat#038515), *ChatCre* (Cat#006410) and Ai9 (Cat#007909) mouse lines were purchased from The Jackson Laboratory (Bar Harbor, ME), while *Glra1^flox/flox^* mice (Cat#T059670) were bought from GemPharmatech (San Diego, CA). All procedures used in this study were approved by the National Institute on Alcohol Abuse and Alcoholism (NIAAA) Animal Use and Animal Care Committee (Protocol No.LIN-LZ-1) and performed in accordance with the National Institutes of Health (NIH) guidelines for care and use of laboratory animals.

### Behavioral tests

Mice were given 1 h for habituation to the environment before each behavioral test. All tests were performed between 10:00 am and 3:00 pm. Mice were 8-10 weeks old at the beginning of behavioral tests.

### Acoustic startle test

Startle responses were detected using SR-LAB test stations and software (San Diego Instruments, San Diego, CA). A protocol adapted from the previous publication from our laboratory was used to generate an Input/Output (I/O) function which represents the correlation between acoustic stimulus intensity and startle response amplitude 3. Mice were habituated to the testing chambers and acoustic stimuli prior to I/O function testing. The acoustic stimuli were 200 ms white noise bursts with <1 ms rise times that varied in decibel intensities. Each trial began with a 4-min baseline period, followed by the presentation of one noise burst after a randomized interval between 75 and 90 s. The stimulus started at 75 dB and increased between each presentation by 5 dB until reaching 120 dB. A 75 dB noise burst was presented 4 times, of which the average response was taken as the baseline (Vb), and other noise bursts were presented only once. The startle response identified as the maximum startle amplitude (Vm) is quantified based on the analog signals transduced from the vibrations induced by whole-body startle reflexes occurring in the Plexiglas cylinder recorded by a piezoelectric unit attached to the platform in the duration of 200 ms following the stimulation. The startle response level is calculated by each response normalized to the baseline (Vm/Vb).

### Tactile startle test

The Plexiglas cylinder of SR-LAB test station was connected to a compressed air cylinder, which allows delivery of an air puff to the mouse's back. The tactile stimuli of 200-ms air puff were given at 1.5 or 5 PSI. A testing session began with a 4-min acclimation period, followed by the presentation of 6 air puff stimuli and then by stimulus deliveries at a random interval between 75 and 90 s. The startle response was defined as the response induced by each air puff to that with no stimulation, and the average of 6 trials was used for further analysis in this test.

### Locomotor activity test

Locomotion was monitored using the VersaMax Animal Activity Monitoring System (Accuscan Instruments Inc., Columbus, OH), interfaced with a computer. Mice were placed into the center of a 45 cm × 45 cm × 40 cm box and allowed to explore the open field for 60 min. Locomotion was detected by disturbance of intersecting photocell beams evenly distributed along the walls. Data was collected in 1-min intervals. The average number of crossed beams in horizontal and vertical dimensions were calculated to assess their locomotor activity.

### Tail flick test

The baseline latency was measured 3 times using the Ugo Basile system (Gemonio VA, Italy), and the mean of the latencies to withdraw the tail was used as the reaction time for each mouse. The light intensity causing 3-7 s baseline latency was used. A cutoff time of 16 s was adopted to avoid tail tissue damage.

### Rotarod test

The protocol was modified from our previous study 46. A computer-interfaced rotarod instrument (ENV-575M, MED Associates, Fairfax, VT) accelerating from 4 to 40 rotations per min over 300 s was used. Mice were trained using 4 trials per day with a 30-min intertrial interval for 4 consecutive days. Each trial ended when the mouse fell off the rotarod or after 300 s had elapsed. The time that each mouse maintained its balance on the rotating rod was measured as the latency to fall. Mice that failed to stand on the rotarod longer than 250 s after 3 days of training were defined as disqualified. On the 4^th^ day, each mouse was placed on the rotarod to test and to record the latency. There was a 15-min interval between 2 consistent trials in this experiment. The average value for a total of 5 trials was used to quantify their motor coordination for analysis.

### Dynamic measurement of metabolic parameters

The protocol for dynamically measuring metabolic parameters was described previously 47. An Oxymax Metabolic Cage System bought from Columbus Instrument (Columbus, OH) was used to indirectly measure metabolic parameters. The system was placed in a specially designed room to avoid disturbance. The system is a non-invasive instrument directly monitoring respiratory exchange ratio (O_2_/CO_2_) and mouse physical activity measured by infrared beams and detectors. The experimental mice were singly housed, and only littermate mice from the same cage were used for metabolic parameter measurement. Then, the real-time metabolic parameters, including respiratory quotient and ambulatory movements, were automatically recorded by the system for 3 complete days. During measurement, mice had access to water and food *ad libitum*.

### Body Temperature Measurement

Experimental mice were subcutaneously implanted with sterile IPTT-300 Implantable Programmable Temperature transponders (Bio Medic Data Systems, Seaford, DE). Before body temperature recording, mice were brought into the procedure room and allowed to habituate for 1 hour. Then, mouse body temperature was measured using an IPTT-300 Implantable Programmable Temperature reader (DAS-8027) in the home cages.

### Stereotaxic injection

Mice were anesthetized with 5% isoflurane and secured in a Kopf stereotaxic frame. Anesthesia was maintained with 1.5-1.8% isoflurane, and mice were placed on a heating pad to prevent hypothermia. An incision at the midline of skull was made using a blade, after the incision site was shaved and disinfected with 75% ethanol. Adeno-associated virus (AAV) was bilaterally injected into the RtTg (anterior-posterior -4.3 mm, medial-lateral ±0.47 mm, dorsal-ventral -4.75 mm) of the brainstem at 200 nL/side (titer: 1.12 × 10^13^ GC/mL) at a speed of 1.5 nL/s with a 10-μL microsyringe (Hamilton, USA). The injector was kept for 10 min after the injection to prevent fluid back flow, and then slowly withdrawn. The incision was closed with tissue glue. Here, *AAV9-CMVCre-EGFP* (using *AAV9-CMV-EGFP* as the control) and *AAV9-Camk2aCre-EGFP* (using *AAV9-Camk2a-EGFP* as the control) were used to induce the deletion of GlyRα1 in the RtTg. The mice with surgery were given 3 weeks for recovery and viral expression.

### Intra-spinal injection

Mice were anesthetized and maintained with 1.5-1.8% isoflurane. The spine was stabilized using a spinal cord clamp (CravitySci, New Castle, DE). After shaving and disinfecting the surgical area with 75% ethanol, an incision was made to expose the spinal cord. To induce cell-specific spinal deletion of GlyRα1, a total of 3 injections of *AAV9-Camk2aCre-EGFP* or* AAV9-CMVCre-EGFP* were infused into the spinal cord at the L3-L4 and L4-L5 levels in GlyRα1 flox mice. Viral injections were delivered at a depth of 200-400 µm using a glass capillary, with a volume of 500 nL per injection (titer: 2.56 × 10^13^ GC/mL). The mice underwent startle tests 3 weeks after the viral injections.

### Jess capillary electrophoresis-based protein quantification

Tissue samples were taken from various mouse brain areas as indicated in the main text for measurement of targeted proteins. Protein separation and analysis of protein density were performed using a Jess automated size-based capillary system (Sally Sue Instruments) from ProteinSimple (San Jose, CA). Briefly, mouse brain was removed after euthanasia and immersed in ice-cold 1× PBS with protease inhibitor cocktail (ThermoFisher Scientific, Waltham, MA). The protein was extracted using M-Per buffer containing protease inhibitor cocktail (ThermoFisher Scientific). Total protein concentrations were measured using a Pierce BCA Protein Assay Kit. The lysate was then diluted using 0.1× sample buffer at a final concentration of 0.3 mg/mL for chemiluminescence. Protein expression was quantified with an automated size-based capillary electrophoresis system (ProteinSimple). The procedures were set as follows: separation time of 25 min with separation voltage of 375 V followed by antibody diluent for 5 min, primary antibody time of 60 min and secondary antibody time of 30 min. The protein expression levels were quantified by integrating the electropherogram peak corresponding to the designated signals. The information about the primary and secondary antibodies is listed in Key Resources Table. The chemiluminescence signals were automatically converted to electropherograms in Compass software (ProteinSimple, version 3.0). Protein expression was quantified by integrating the electropherogram peak corresponding to the signal.

### RNA isolation and real-time qPCR

Total RNA was extracted from the tissues of mouse spinal cord and brainstem using mini RNeasy kit (Qiagen) following the manufacturer's instructions. A total of 1 μg of RNA was reverse transcribed into complementary DNA (cDNA) using the High-Capacity cDNA Reverse Transcription kit (ThermoFisher Scientific). Real-time quantitative PCR (qPCR) was performed using the SYBR Green Realtime PCR master mix (ABM, BlasTaq 2× qPCR, Cat#G892). The expression levels of target genes were normalized to Beta-Actin RNA expression. The comparative Ct (2^-ΔΔCt^) method was performed to quantify the mRNA expression level. All primers used for real-time qPCR are listed on the Key resource table (Table 1).

### RNAscope *in situ* hybridization

RNAscope fluorescent *in situ* hybridization was carried out according to the RNAscope Fluorescent Multiplex Reagent Kit User Manual or Ascope HiPlex v2 User Manual [Advanced Cell Diagnostics (ACD), Newark, CA]. Mouse brain tissues were quickly removed and frozen on dry ice after euthanasia. Fresh-frozen tissue sections (14 μm) were cut using a cryostat (CM3050S, Leica Biosystem, Nussloch, Germany) and mounted on positively charged microscopic glass slides. Slides are stored at -80 °C for further use. The information about the probes and kits used is listed in the Key resource table (Table 1).

### RNAscope Multiplex assay

Slides were immersed in 4% paraformaldehyde (PFA) for post-fixation at 4 °C for 60 min, then washed twice in 1x PBS and treated by 50%, 70% and 100% ethanol in turn at room temperature (RT) for 5 min each. Subsequently, the slides were treated with hydrogen peroxide for 10 min and digested with protease IV for 20 min at RT. The slides were washed with 1x PBS three to five times, incubated with probes and placed in an incubation oven (HybEZ II, ACD) at 40 °C for 2 h. The slides were washed twice with 1x wash buffer at RT and then hybridized with FLv2 Amp1, Amp2 or Amp3 and labelled with FLv2 HRP C1, C2 or C3 signals. All slides were blocked by FLv2 HRP blocker followed by DAPI treatment for 30 s. The slides were cover-slipped with Prolong Gold Antifade fluorescence mounting medium (ThermoFisher Scientific) and sealed for imaging. Epifluorescence images were obtained through a Zeiss Axiozoom microscope (Carl Zeiss, Oberkochen, Germany) equipped with standard Cy3 filters via a Zeiss Axiocam MR monochrome CCD.

### RNAscope Hiplex assay

Slides were immersed in 4% PFA at 4 °C for 60 min, then washed twice in 1x PBS and treated by 50%, 70% and 100% ethanol in turn at RT for 5 min each. The slides were then digested with protease IV for 30 min at RT. The slides were washed with 1x PBS three to five times, incubated with the customized probes (T1-T9) and incubated at 40 °C for 2 h. The slides were washed twice with wash buffer and hybridized with HiPlex Amp1, Amp2 or Amp3 to magnify the signals. Then the slides were hybridized with Fluoro T1-T3 for 40 °C for 15 min and washed twice in 1x wash buffer for 2 min each. Slides were treated with DAPI treatment for 30 s and mounted with Prolong Gold Antifade fluorescence mounting medium but not sealed. The slides were imaged for Round 1. After being imaged, the slides were soaked in 4x SSC solution at RT for 1 h to remove the coverslip, briefly washed once in 4x SSC and incubated with 10% cleaving solution at RT for 15 min. Wash once with PBST (1x PBS containing 0.5% Tween), and repeat the cleaving procedure. Then the slides were hybridized with Fluoro T4-T6 for 40 °C for 15 min and washed twice in 1x wash buffer for 2 min each. The slides were then mounted and imaged for Round 2. Repeat the procedures of Round 2 to hybridize Fluoro T7-T9 and image the slides for Round 3. The identical areas were imaged in each Round. Finally, the images were merged with Hiplex Image Registration software (ACD).

### Pressure-volume catheterization

Heart left ventricular performance was assessed using the P-V method, as described 48. A pressure-volume catheter connected to a 1.4 F size microtip (SPR-839; Millar Instruments, Houston, TX, USA) was inserted into the left heart ventricle (LV) via the right carotid artery. Polyethylene cannulas (PE-10) were secured in the left jugular vein for drug delivery. P-V signals were recorded using an AD Instruments PowerLab data acquisition system (Colorado Springs, CO, USA) after it was fully stabilized. The major cardiac contractile indices were adopted, including the end-systolic pressure (ESP), the maximal slope of systolic pressure increment (dP/dtmax), stroke work (SW) cardiac output, ejection fraction (EF), stroke volume and heart rate. Gradual preload reduction induced by vena cava occlusion was taken to calculate the load-, and heart rate-independent parameters of left ventricular performance, such as the ventricular end-systolic elastance (Ees), preload recruitable stroke work (PRSW), and the dP/dtmax -end-diastolic volume (EDV) relationship as previously described 49. Cross-sectional areas of cardiac muscle sections were also measured in this test.

### Spinal cord slice electrophysiological recording

Whole-cell electrophysiological recordings in spinal cord slices were performed as reported previously 3, 50. Mice were anesthetized via inhalation of 3% isoflurane, and the lumbar spinal cords were quickly removed via laminectomy. The spinal cords were immerged in ice-cold sucrose-modified artificial cerebrospinal fluid containing (in mM) 234 sucrose, 26 NaHCO_3_, 1.2 NaH_2_PO_4_, 3.6 KCl, 2.5 CaCl_2_, 1.2 MgCl_2_, and 25 glucose (gassed with 95% O_2_ and 5% CO_2_), and 400-μm thick transverse spinal cord slices were sectioned using a vibratome (Leica Biosystems, Nussloch, Germany). Slices were then preincubated in the 95% O_2_ and 5% CO_2_ oxygenated Krebs solution containing (in mM) 117 NaCl, 25 NaHCO_3_, 1.2 NaH_2_PO_4_, 3.6 KCl, 2.5 CaCl_2_, 1.2 MgCl_2_, and 11 glucose, at 34 °C for at least 1 h before recording.

Spinal cord slices were fixed onto a glass-bottomed recording chamber and continuously perfused with oxygenated Krebs solution at 3 ml/min at 34°C. The tdTomato-labeled neurons in the spinal cord lamina II were identified using a combination of epifluorescence illumination and infrared and differential interference contrast optics on an Olympus upright microscope 51. Whole-cell voltage-clamp recordings were performed using borosilicate glass electrodes (5-8 MΩ) filled with the internal solution containing (in mM) 110 Cs_2_SO_4_, 2 MgCl_2_, 0.5 CaCl_2_, 10 HEPES, 5 EGTA, 2 Mg-ATP, 0.3 Na_2_-GTP, and 10 QX314 (280-300 mOsm, pH 7.3). We recorded glycine-elicited currents at a holding potential of +10 mV by puff application of 1 mM glycine onto the labeled neuron at distance of 150 μm using a positive pressure system (Toohey Spritzer, Toohey Company, NJ). We also recorded GABA-elicited currents by puff application of 100 μM GABA at the holding potential of +10 mV 52, 53.

Spontaneous excitatory synaptic currents (sEPSCs) were recorded at a holding potential of -60 mV, and spontaneous inhibitory synaptic currents (sIPSCs) were recorded at a holding potential of +10 mV. Glycinergic sIPSCs (Gly-sIPSCs) were recorded in the presence of 10 μM gabazine, a specific GABA_A_ receptor antagonist. GABAergic sIPSCs (GABA-sIPSCs) were recorded in the presence of 2 µM strychnine, a specific glycine receptor antagonist 53. Signals were filtered at 1-2 kHz and digitized at 10 kHz using a DigiData 1320A and a MultiClamp 700B amplifier (Molecular Devices, CA). The input resistance was monitored, and the recording was abandoned if it changed >15%. Only 1 neuron was recorded from each spinal cord slice. The amplitude of puff GABA and glycine currents was quantified by averaging 3 consecutive traces using Clampfit 11 software (Molecular Devices, CA). sEPSCs and sIPSCs were analyzed using the MiniAnalysis program (Synaptosoft, GA) over a recording period of 3 min. At the end of the recording, 2 µM strychnine, 10 μM gabazine, or 10 µM DNQX, a glutamate AMPA receptor antagonist, was bath-applied to validate that the currents were mediated by glycine, GABA_A_ or AMPA receptors 54. All drugs were freshly prepared in artificial cerebrospinal fluid before recording and delivered at their final concentrations using syringe pumps.

### RNA-sequencing analysis

Total RNA was extracted from the tissues of mouse spinal cord and brainstem with QiaGen RNeasy mini kit (Germantown, MD). RNA-sequencing analysis was carried out using the Illumina NovaSeq 6000 platform (San Diego, CA). Paired-end clean reads were aligned to the mouse reference genome (Ensemble_GRCm38.90) with TopHat (Version 2.0.12), and HTSeq-count (Version 0.6.1) was used to quantify the mRNA level. The criteria P value < 0.01 and |Log_2_Fold Change|>0.5 were set to screen differentially expressed genes (DEGs) from RNA-Seq transcriptome. DEGs were selected to conduct Gene Ontology (GO) analysis.

### Statistical analysis

GraphPad Prism 11.0 (San Diego, USA) was utilized to perform statistics and plot figures in this study. ZEN Microscopy Software 2.3 (Zeiss, Oberkochen, Germany) was used to visualize stained tissues, while ImageJ (NIH, USA) was adopted to count cells and signals. For 2-group comparisons, unpaired Student's *t* test was carried out if the data met normal distribution criteria checked by the Shapiro-Wilk test, otherwise the Mann-Whitney test was used to assess the differences. To compare the difference among 3 groups or more, ANOVA (One-way or Repeated measures) followed by Tukey's multiple comparison tests was performed. Kolmogorov-Smirnov analysis was also performed to analyze the data from whole-cell clamp patch recordings. A significant difference was set as p < 0.05. Data are presented as the mean±SEM.

## Supplementary Material

Supplementary figures.

## Figures and Tables

**Table 1:** Key resource table.

Reagents or Resources	Source	Identifier or Sequence
**Antibodies or Reagents**		
Rabbit anti-GlyRα1 antibody	Novus Biologicals	Cat#NB300-113
Mouse HRP anti-beta actin antibody [AC-15]	Abcam	Cat#ab49900
Anti-rabbit secondary antibody	Bio-Techne	Cat#108461
Prolong Gold Antifade Reagent	ThermoFisher Scientific	Cat#P36930
**RNAscope probes**		
**Multiplex assay**		
Mm-Camk2a-C1	Bio-Techne	Cat#445231-C1targeting 896-1986 of NM_009792.3
Mm-Camk2a-C2	Bio-Techne	Cat#445231-C2 targeting 896-1986 of NM_009792.3
Mm-Camk2a-C3	Bio-Techne	Cat#445231-C3 targeting 896-1986 of NM_009792.3
Mm-Chat-C1	Bio-Techne	Cat#408731-C1 targeting 1090-1952 of NM_009891.2
Mm-Chat-C2	Bio-Techne	Cat#408731-C2 targeting 1090-1952 of NM_009891.2
Mm-Glra1-C1	Bio-Techne	Cat#506061-C1 targeting 1000-2413 of NM_001290821.1
Mm-Glra1-C3	Bio-Techne	Cat#506061-C3 targeting 1000-2413 of NM_001290821.1
Mm-Fos-C1	Bio-Techne	Cat#316921-C1 targeting 407-1427 of NM_010234.2
Mm-Fos-C2	Bio-Techne	Cat#316921-C2 targeting 407-1427 of NM_010234.2
Mm-Slc6a5-C2	Bio-Techne	Cat#409741-C2 targeting 925-2153 of NM_148931.3
Mm-Slc17a6-C1	Bio-Techne	Cat#319171-C1 targeting 1986- 2998 of NM_080853.3
Mm-Slc17a6-C2	Bio-Techne	Cat#319171-C2 targeting 1986- 2998 of NM_080853.3
Mm-Slc17a6-C3	Bio-Techne	Cat#319171-C3 targeting 1986-2998 of NM_080853.3
Mm-Egfp-C1	Bio-Techne	Cat#400281-C1 targeting 628-1352 of U55763.1
**Hiplex assay**		
Mm-Camk2a-T3	Bio-Techne	Cat#445237-T3 targeting 896-1986 of NM_009792.3
Mm-Chat-T4	Bio-Techne	Cat#408731-T4 targeting 1090-1952 of NM_009891.2
Mm-Slc17a6-T5	Bio-Techne	Cat#319171-T5 targeting 1986-2998 of NM_080853.3
Mm-Slc6a5-T8	Bio-Techne	Cat#409741-T8 targeting 925-2153 of NM_148931.3
Mm-Glra1-T9	Bio-Techne	Cat#506061-T9 targeting 1000-2413 of NM_001290821.1
**Chemicals**		
glycine	MilliporeSigma	Cat#G7126
GABA	MilliporeSigma	Cat#A2129
strychnine	MilliporeSigma	Cat#S8753
DNQX	Abcam	Cat#ab120169
gabazine	MilliporeSigma	Cat#S106
**qPCR primers**		
Mouse Glra1-Forward	QIAGEN	AGGACTTCTGGGTATGACGCCA
Mouse Glra1-Reverse	QIAGEN	GTTGTCTCAGCGATGGAACCGA
Mouse ACTB-Forward	QIAGEN	GTGCTATGTTGCTCTAGACTTCG
Mouse ACTB- Reverse	QIAGEN	ATGCCACAGGATTCCATACC

## Abbreviations

GlyRα1: glycine receptor alpha 1
GlyT2: glycine transporter type 2
ChAT: choline acetyltransferase
CamK2α: calcium/calmodulin dependent protein kinase II alpha
vGluT2: vesicular glutamate transporter 2
KO: knockout
