# Supplementary material for: Spinal glycine receptor alpha 1 coordinates startle behavior through a cell-type specific mechanism
Source: Int J Biol Sci. 2026 Jul 13;22(12):6631–52. doi: 10.7150/ijbs.132688 (PMC13411978; doi:10.7150/ijbs.132688)
Supplement: Supplementary file 1 — Supplementary figures. [file ijbsv22p6631s1.pdf]

Supplementary figures

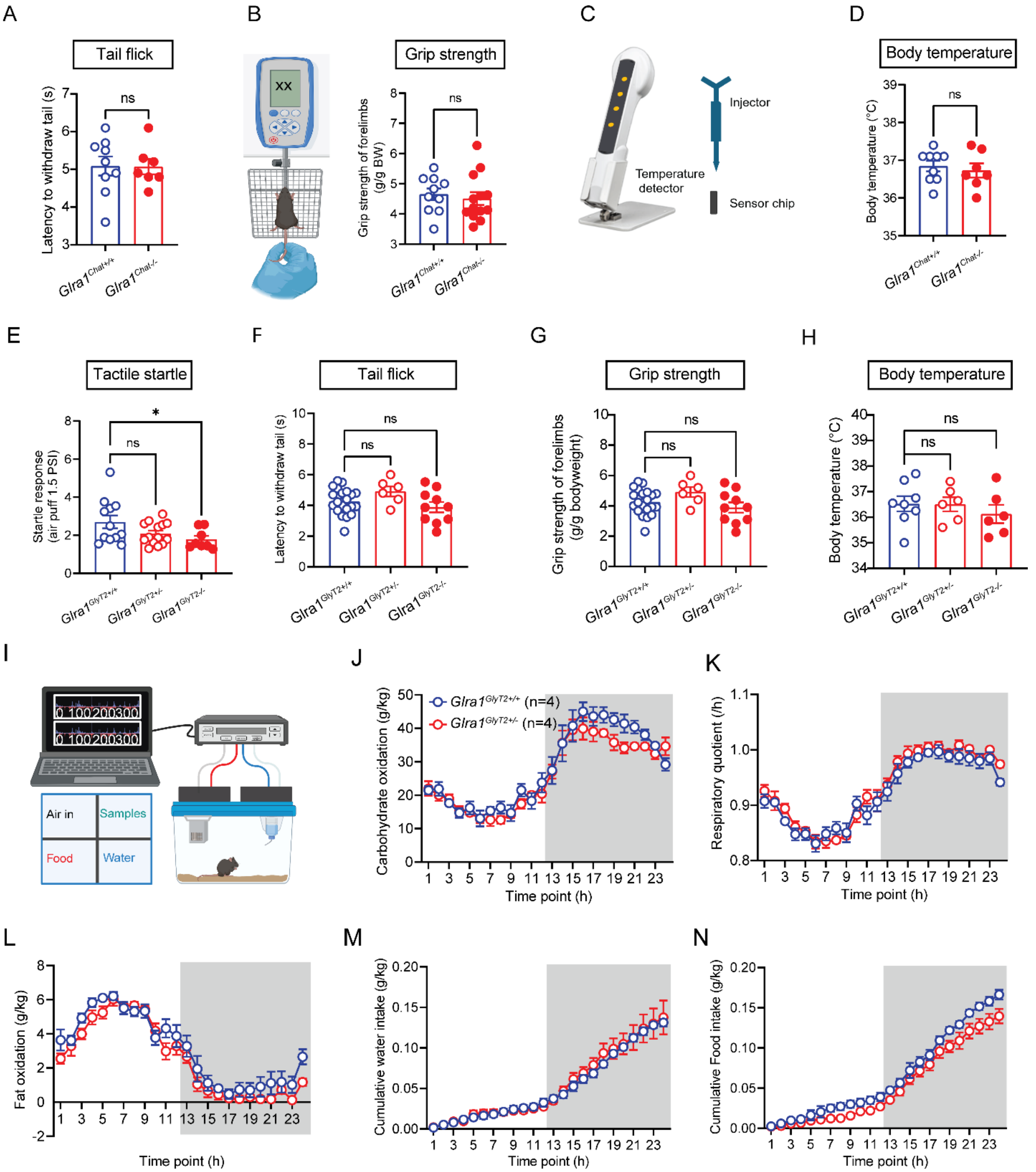

Supplementary Figure 1 No alterations in pain threshold, forelimb grip strength, body temperature or metabolic parameters in *Glra1*$^{Chat-/-}$ and *Glra1*$^{GlyT2-/-}$ mice. (A-D) There were no differences in pain threshold in the tail flick test, forelimb grip strength or

body temperature in *Glra1*$^{Chat-/-}$ mice compared to *Glra1*$^{Chat+/+}$ mice. **(E)** The startle responses to air puff at 1.5 PSI did not significantly differ between *Glra1*$^{GlyT2+/-}$ and *Glra1*$^{GlyT2+/+}$mice. **(F-H)** There were no differences in pain threshold, forelimb grip strength or body temperature in *Glra1*$^{GlyT2-/-}$ mice compared to *Glra1*$^{GlyT2+/-}$ and *Glra1*$^{GlyT2+/+}$ mice, respectively. **(I-N)** Selective GlyRα1 deletion from GlyT2-expressing neurons did not change carbohydrate oxidation, respiratory quotient, fat oxidation, cumulative water or food intake in mice, evaluated with the metabolic measurement cages (n= 4 /group).

One-way ANOVA or unpaired *t* test was used, as appropriate. *p < 0.05; ns, not significant. Error bars indicate mean±SEM.

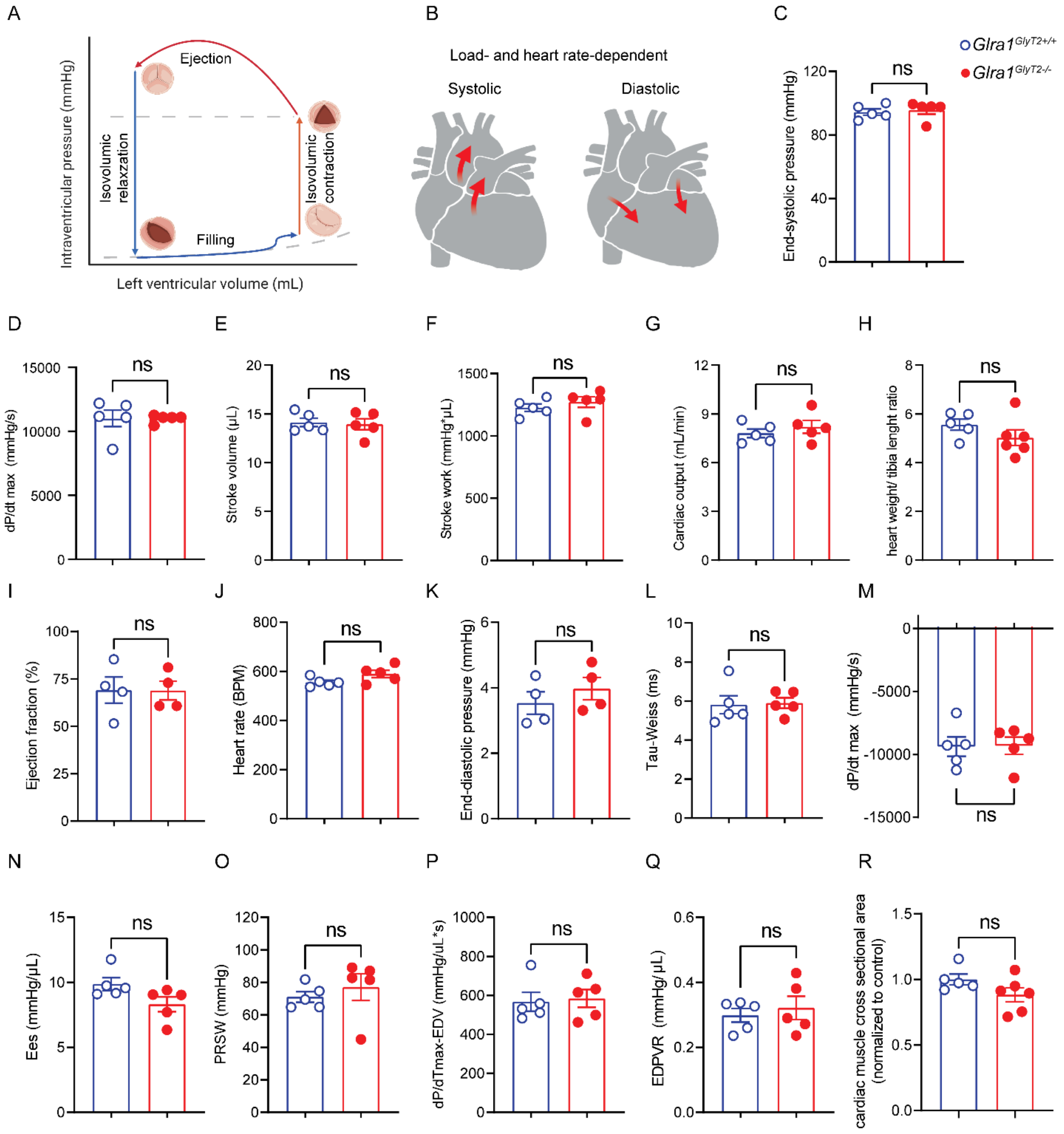

Supplementary Figure 2 No alterations in the left ventricular function in *Glra1*$^{GlyT2-/-}$ mice. (A) A pressure-volume (PV) loop showing the heart's mechanical performance during a cardiac cycle. **(B)** Schematic of systolic and diastolic function. **(C-J)** There were no alterations in systolic functions dependent on load and heart rate, including end-systolic pressure, dP/dt max, stroke volume, stroke work, cardiac output and ejection fraction **(C-G, I)** in *Glra1*$^{GlyT2-/-}$ mice (n = 5), compared to the control group (n = 5). Similarly,

there were no alterations in heart rate **(J)** and heart weight/tibia length ratio **(H)** among groups**. (K-M)** There were no alterations in diastolic functions dependent on load and heart rate, including end-diastolic pressure, tau-weiss or -dP/dt max in *Glra1*$^{GlyT2-/-}$ mice, compared to the control. **(N-Q)** There were no alterations in systolic or diastolic functions independent of load and heart rate, including end-systolic elastance (Ees), preload recruitable stroke work (PRSW), dP/dtmax-end-diastolic volume (EDV) or end-diastolic pressure-volume relationship (EDPVR) in *Glra1*$^{GlyT2-/-}$ mice, compared to the control. **(R)** The cardiac muscle cross sectional area was not different between *Glra1*$^{GlyT2-/-}$ and *Glra1*$^{GlyT2+/+}$ mice.

Unpaired *t* test was used. ns, not significant. Error bars indicate mean±SEM.

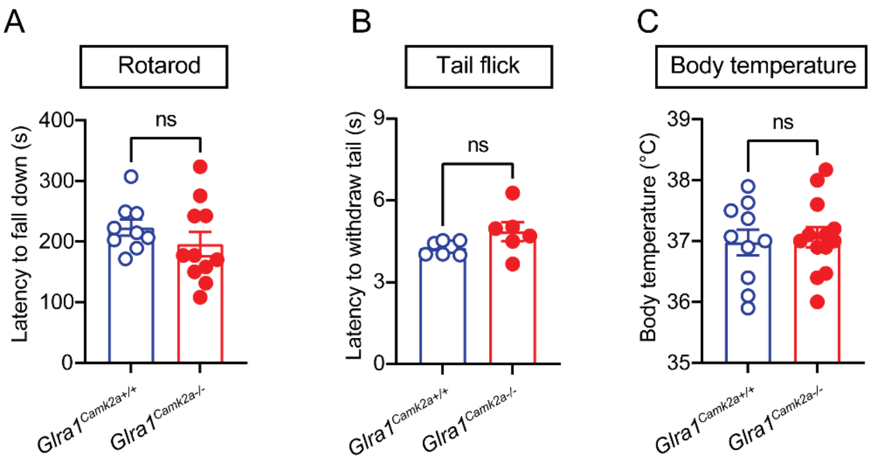

Supplementary Figure 3 No alterations in motor coordination, pain threshold or body temperature in *Glra1*$^{Camk2a-/-}$ mice. (A-B) Conditional deletion of GlyRα1 from CamK2α-positive neurons did not affect the latencies to fall in the rotarod test (n = 10-13 /group) or pain threshold in the tail flick test (n = 6-7 /group). **(C)** The body temperature did not differ between *Glra1*$^{Camk2a+/+}$ (n = 10) and *Glra1*$^{Camk2a-/-}$ mice (n = 13).

Unpaired *t* test was used. ns, not significant. Error bars indicate mean±SEM.

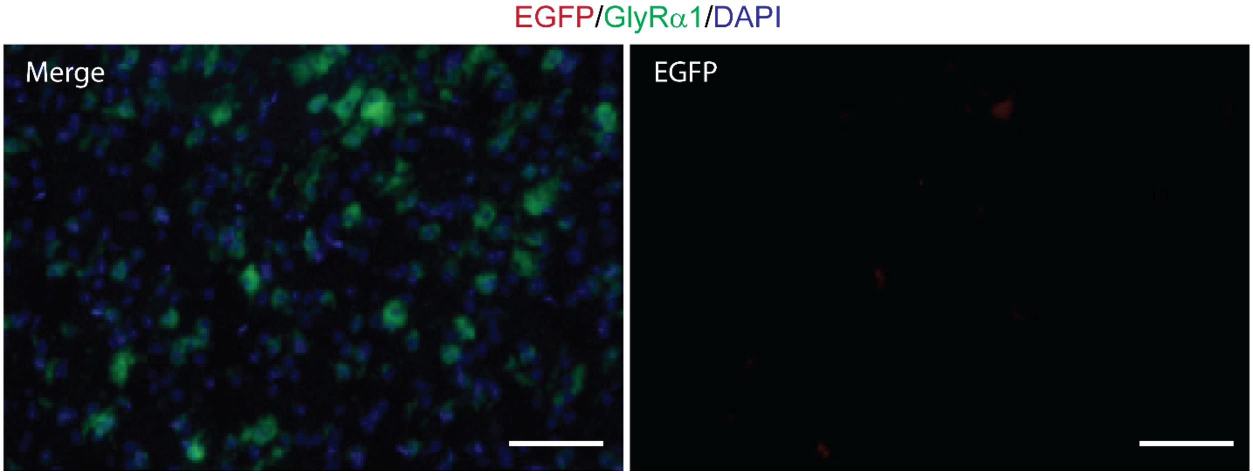

Supplementary Figure 4 The spinally injected AAV did not affect the brainstem. We did not find any EGFP expressed in the brainstem of the mice with spinal injections of *AAV9-CMVCre-EGFP*. Here, the slices of pons were stained with RNAscope assay and shown to represent the brainstem**.** Scale bar: 50 μm.

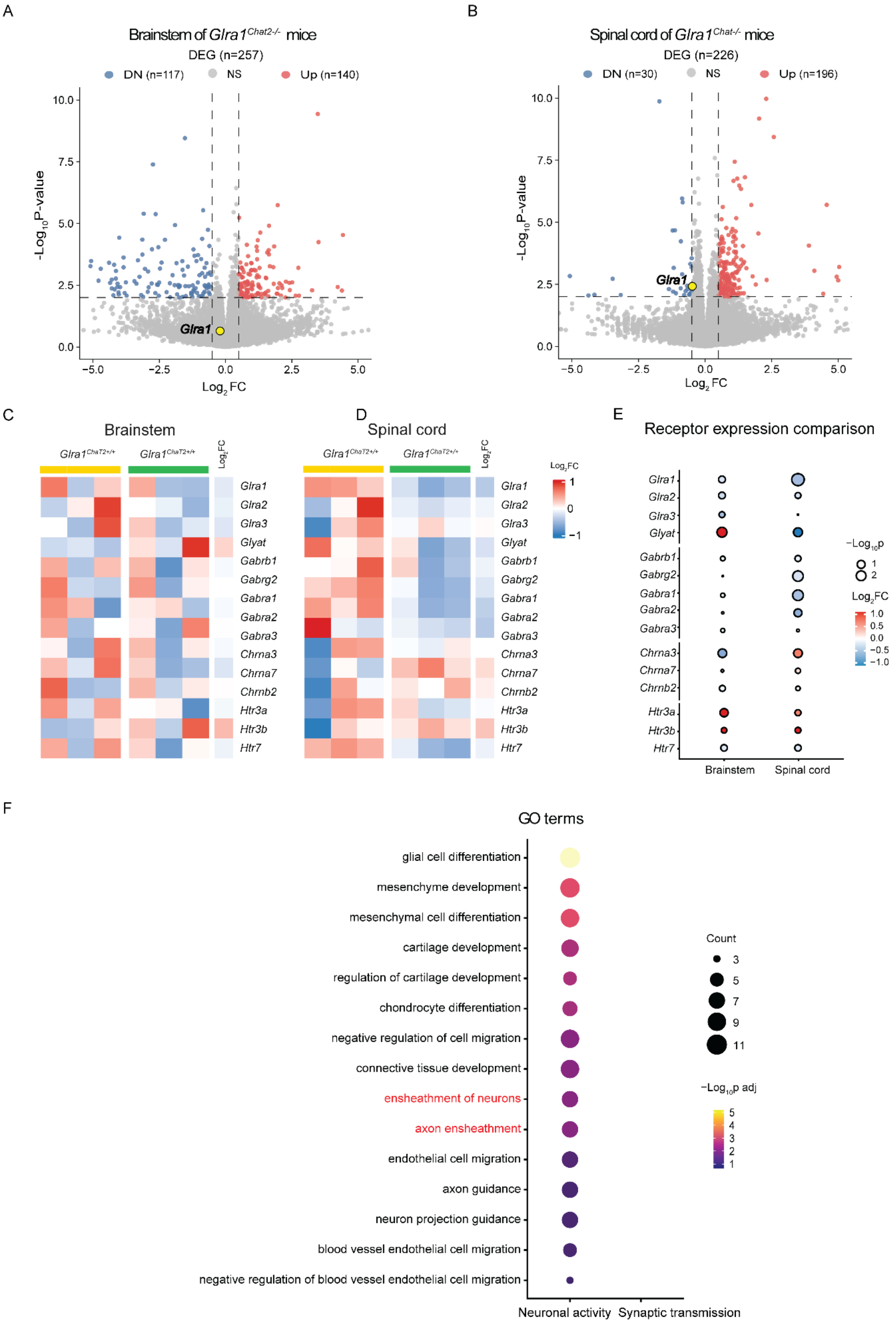
A
Brainstem of Glra1 Chat2-/- mice
DEG (n=257)
DN (n=117) NS Up (n=140)
-Log10 P-value
Log2 FC
Glra1
B
Spinal cord of Glra1 Chat-/- mice
DEG (n=226)
DN (n=30) NS Up (n=196)
C
Brainstem
D
Spinal cord
Log2FC
Glra1
Glra2
Glra3
Glyat
Gabrb1
Gabrg2
Gabra1
Gabra2
Gabra3
Chrna3
Chrna7
Chrnb2
Htr3a
Htr3b
Htr7
E
Receptor expression comparison
Brainstem
Spinal cord
F
GO terms
glial cell differentiation
mesenchyme development
mesenchymal cell differentiation
cartilage development
regulation of cartilage development
chondrocyte differentiation
negative regulation of cell migration
connective tissue development
ensheathment of neurons
axon ensheathment
endothelial cell migration
axon guidance
neuron projection guidance
blood vessel endothelial cell migration
negative regulation of blood vessel endothelial cell migration
Neuronal activity
Synaptic transmission
Count
−Log10p adj

Supplementary Figure 5 RNA-seq analysis: ChAT-GlyRα1 deficiency promotes the changes in gene expression profiles in the spinal cord. (A) Volcano maps showed that the levels of GlyRα1 mRNA did not differ in the brainstem of $Glra1^{Chat-/-}$ mice, while **(B)** the level was significantly decreased in the spinal cord of $Glra1^{Chat-/-}$ mice, compared to $Glra1^{Chat+/+}$ mice. **(C-D)** Heatmaps of gene expression showed that GlyRα1 mRNA level in the spinal cord rather than in the brainstem was significantly reduced, based on the RNA-seq data from the tissues of $Glra1^{Chat+/+}$ and $Glra1^{Chat-/-}$ mice (n = 3 /group). **(E)** The mRNA levels of LGICs in the brainstem and spinal cord were compared between $Glra1^{Chat+/+}$ and $Glra1^{Chat-/-}$ mice, suggesting that the spinal cord showed a more significant change in the level of GlyRα1 mRNA, compared to that in brainstem. **(F)** GO analysis showed that the terms mainly related to "*neuronal activity*" were significantly enriched in the spinal cord of $Glra1^{Chat-/-}$ mice, while the terms related to "*synaptic transmission*" were not significantly enriched in this test.
